# Pleural Mesothelioma: Pathogenesis, Diagnosis, Treatment, Prognosis, and Survival

**DOI:** 10.1002/mco2.70327

**Published:** 2025-09-01

**Authors:** Libo Zhang, Meijuan Huang

**Affiliations:** ^1^ Division of Thoracic Tumor Multimodality Treatment and Department of Medical Oncology Cancer Center West China Hospital Sichuan University Chengdu Sichuan Province China

**Keywords:** chemoresistance, epigenetic modifications, immunotherapy, pleural mesothelioma, targeted therapy

## Abstract

Pleural mesothelioma (PM) presents significant challenges in clinical management, with current treatment options such as chemotherapy, anti‐angiogenic therapies, and immunotherapies only modestly extending progression‐free survival (PFS) and overall survival (OS). Another relevant reason is the absence of subsequent‐line therapy strategies following progression of PM after approved therapy. Despite extensive research efforts, the development of effective targeted therapies has proven difficult, as most identified mutations in PM tend to be tumor suppressors rather than the driving mutations seen in other cancers. This review aims to provide an in‐depth analysis of the biological mechanisms of PM, focusing on genetic alterations, the tumor's immune microenvironment, and dysregulated signaling pathways that contribute to tumorigenesis and resistance to treatment. Additionally, we discuss the growing importance of biomarkers for patient stratification and the development of personalized therapeutic approaches tailored to individual molecular profiles. We also explore promising avenues for novel therapeutic strategies, such as combination therapies and immunotherapeutic interventions. By integrating insights from both basic and clinical research, this review seeks to present a comprehensive framework for understanding PM and advancing its therapeutic management, ultimately aiming to improve patient outcomes through more effective and targeted treatment approaches.

## Graphical abstract

This is an overview of current and emerging therapeutic strategies for pleural mesothelioma (PM), including chemotherapy, immunotherapy (ICIs, dendritic cell vaccines, CAR‐T therapy), anti‐angiogenic therapy, epigenetic modifications (DNA methylation, histone changes, non‐coding RNAs), and targeted therapy focusing on genomic alterations and signaling pathways associated with PM. These approaches represent the evolving landscape of PM treatment.

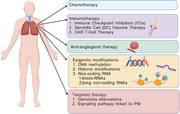

## 1 Introduction

Mesothelioma is a rare malignancy originating from the serosal membranes, with the pleura being the predominant site of manifestation. Despite its relatively low incidence, this cancer has drawn considerable attention due to its profound association with asbestos exposure [1]. Over the past decade, even with stringent regulations and outright bans on asbestos use in numerous countries, the incidence of pleural mesothelioma (PM) has not markedly decreased, underscoring the prolonged latency period of the disease and the residual impact of historical asbestos exposure [2]. According to the most recent World Health Organization (WHO) classification, PM is delineated into three histological subtypes: epithelioid, sarcomatoid, and biphasic [3]. The epithelioid subtype, which constitutes approximately 70% of cases, is typically associated with a relatively favorable prognosis owing to its less aggressive biological behavior, while the sarcomatoid subtype stands opposite [4].

**FIGURE 1:**
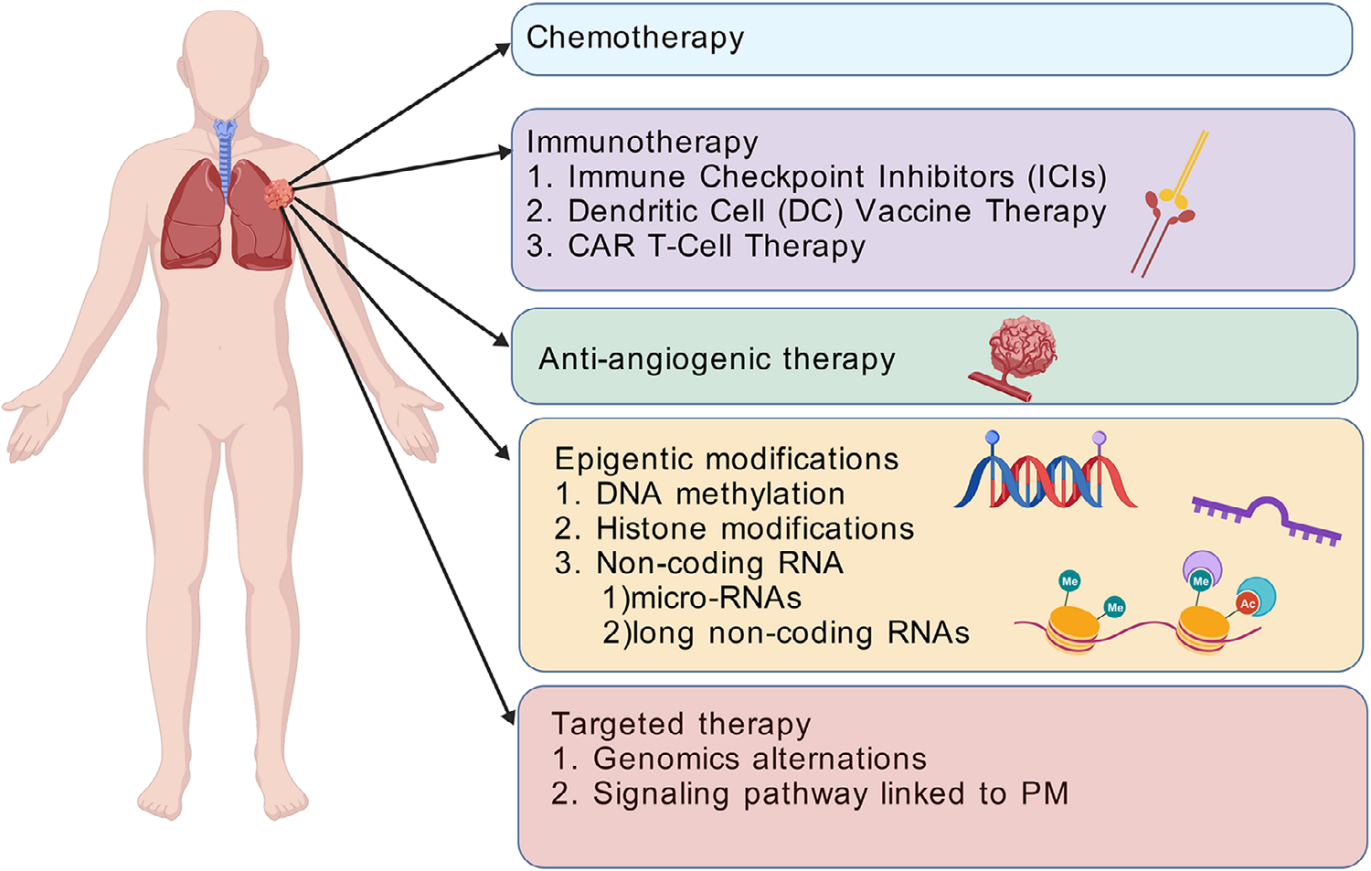
Current treatment strategies for pleural mesothelioma (PM).

Current treatment strategies for PM remain limited. Surgical treatment is one of the main treatment methods for patients with localized PM. For patients with unresectable Stage IIIB–IV PM, the first‐line treatment strategy has undergone three pivotal advancements. The first significant development occurred in 2003 when pemetrexed combined with cisplatin was established as the global standard of care for PM [5]. The second milestone was achieved in 2015, when it was demonstrated that adding bevacizumab, an anti‐angiogenic agent, to pemetrexed and cisplatin significantly improved overall survival (OS), compared to standard chemotherapy [6]. The third major development emerged in 2020 with the advent of immunotherapy, marking a new era in PM treatment. The CheckMate 743 (CM‐743) trial confirmed the efficacy of nivolumab combined with ipilimumab, a dual immunotherapy regimen that conferred a significant survival advantage for PM patients [7] (Figure 1).

Although surgery, conventional chemotherapy, and existing immunotherapies have demonstrated certain efficacy, the high recurrence rates and resistance associated with the disease continue to result in poor patient prognosis. Concurrently, with the continuous emergence of innovative therapeutic modalities such as immunotherapy and targeted treatment, the challenge now lies in how to strategically integrate these novel approaches and devise individualized treatment regimens. This has become a focal point of contemporary research and clinical practice. This review addresses the key challenges and advancements in PM research, focusing on drug resistance mechanisms, novel therapeutic agents, clinical trials, and the role of epigenetic and targeted therapies. We highlight the latest insights into PM's molecular landscape and discuss innovative treatment strategies, including selective inhibitors, dual‐target therapies, and epigenetic modulators, aimed at improving clinical outcomes and translating research into effective treatments.

## 2 Pathogenesis

**FIGURE 2:**
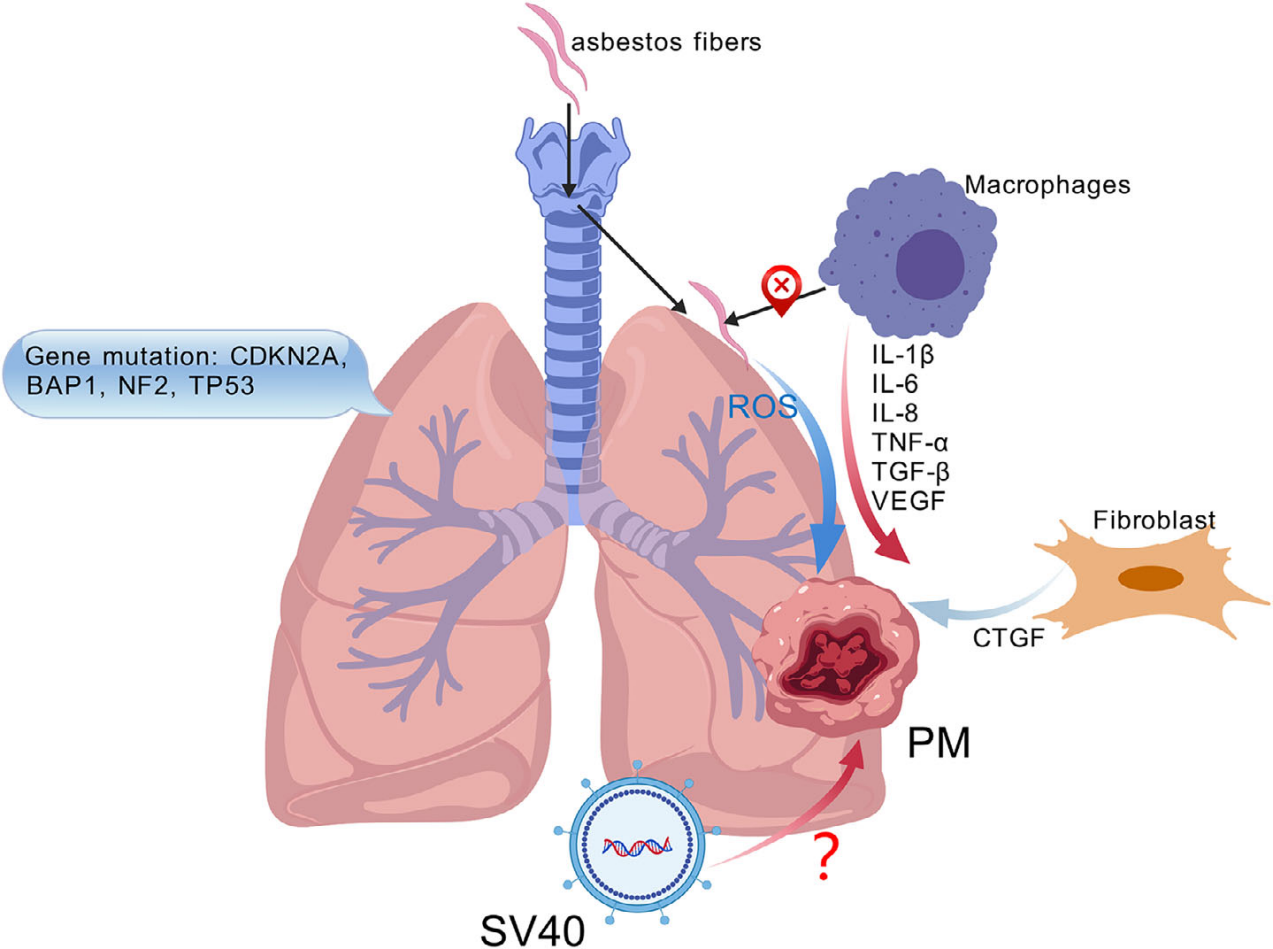
Pathogenesis of PM. ROS: reactive oxygen species; CTGF: connective tissue growth factor; SV40: Simian virus 40.

PM arises from the intricate interplay of environmental, genetic, and biological factors; however, its precise pathogenesis remains incompletely understood. Predominantly, chronic occupational exposure to asbestos fibers initiates the pathogenic cascade through interference with mitotic processes, induction of reactive oxygen species (ROS), and the activation and accumulation of macrophages, thereby driving persistent mesothelial inflammation and subsequent malignant transformation [8] (Figure 2). The pathogenesis of PM is characterized by asbestos‐induced mesothelial cell necrosis triggering the release of HMGB1, which recruits macrophages to secrete pro‐inflammatory cytokines (e.g., IL‐1β), establishing a chronic inflammatory microenvironment that drives malignant transformation [9], triggering their secretion of pro‐inflammatory cytokines, such as IL‐1β, thus establishing a chronic inflammatory microenvironment conducive to malignancy. This chronic milieu perpetuates the secretion of numerous pro‐inflammatory and angiogenic mediators, including IL‑1β, IL‑6, IL‑8, TNF‑α, TGF‑β, and VEGF, creating an environment characterized by heightened oxidative stress, pronounced angiogenesis, and pronounced immune suppression [10, 11, 12]. The inflammatory microenvironment recruits and polarizes macrophages, facilitating pleural fibrosis and extracellular matrix (ECM) remodeling driven predominantly by connective tissue growth factor (CTGF)‐expressing fibroblasts. Concurrently, natural killer (NK) cells and cytotoxic CD8⁺ T lymphocytes exhibit compromised effector functions, while immunosuppressive activities of CD4⁺ T cells and macrophages are significantly enhanced [13, 14, 15]. This sustained oxidative damage, coupled with a cytokine‐driven inflammatory cascade and progressive fibrosis, constitutes a deleterious cycle promoting mesothelial malignancy, tumor invasiveness, and resistance to immunotherapy, thereby critically influencing disease progression.

Additionally, impairments in DNA repair mechanisms and tumor suppressor genes are intimately linked to mesothelioma pathogenesis. Approximately 10% of mesothelioma patients harbor germline mutations in cancer susceptibility genes, predominantly involving CDKN2A, neurofibromatosis type 2 (NF2), and BRCA1‐associated protein 1 (BAP1) [16]. Approximately 35% of germline BAP1 mutation carriers develop mesothelioma, typically asbestos‐independent, early‐onset, and slow‐progressing, with nuclear BAP1 loss and papillary features [17]. Termed low‐grade BAP1‐associated mesothelioma (L‐BAM), this indolent subtype may benefit from conservative management distinct from that of aggressive sporadic disease. Deletion of CDKN2A on chromosome 9 disrupts pRB‐ and p53‐mediated cell cycle checkpoints, enhancing tumor aggressiveness and invasiveness [18]. Mutations in BAP1 result in loss of ubiquitin hydrolase activity and defective nuclear localization, profoundly affecting critical pathways including DNA damage repair, cell cycle progression, and apoptosis. These mutations are frequently observed in PM and are paradoxically associated with improved long‐term survival when present in germline form [19, 20, 21]. Moreover, NF2 inactivation compromises Merlin protein function, aberrantly activating the Hippo and mechanistic target of rapamycin (mTOR) signaling pathways, thus substantially promoting cellular proliferation, migration, and invasion, particularly pronounced in highly aggressive sarcomatoid mesothelioma [22]. Furthermore, TP53 mutations and functional loss similarly accelerate tumor progression, notably resulting in highly invasive mesothelioma subtypes predominantly affecting young female patients and correlating with poor clinical outcomes [23].

Simian virus 40 (SV40), a DNA polyomavirus originally isolated from monkey kidney cells, exhibits potent oncogenicity in experimental models. Its large T antigen exerts transformative effects by functionally inactivating key tumor suppressors, including p53 and Rb. Owing to this oncogenic potential, early studies reported the presence of SV40 DNA sequences and T antigen expression in PM tissues, leading to the hypothesis that SV40 might act synergistically with asbestos exposure to promote tumorigenesis [24]. However, more recent investigations employing highly specific detection techniques—such as RNAscope, low‐contamination‐risk PCR primers, and immunohistochemistry—have uniformly failed to detect SV40 large T antigen in extensive cohorts of PM cases from regions with high asbestos exposure, including the United Kingdom and Dayao County in Yunnan, China. These findings suggest that earlier reports of SV40 positivity may reflect technical artefacts or laboratory contamination [25, 26]. Moreover, epidemiological data indicate that despite the widespread administration of SV40‐contaminated polio vaccines in the mid‐20th century, no corresponding increase in mesothelioma incidence has been observed in affected populations [27, 28]. Collectively, the current evidence does not support a causal role for SV40 in PM pathogenesis. Asbestos exposure remains the predominant and most well‐established etiological factor.

## 3 Diagnosis

The hallmark clinical manifestations of PM predominantly include severe chest pain refractory to analgesics and progressive dyspnea. Additional symptoms such as persistent dry cough, nausea, anorexia, pronounced weight loss over a short period, fever, and nocturnal hyperhidrosis may also occur, with advanced stages often progressing to cachexia. Early diagnosis of PM is critical for enhancing patient survival rates and improving prognosis.

Imaging plays a central role in the diagnosis, treatment planning, and monitoring of PM, with computed tomography (CT) serving as the primary evaluation tool. Chest radiographs lack the sensitivity and specificity required for accurate diagnosis and staging. Large pleural effusions can be obscure. CT is essential for evaluating the extent of PM and distinguishing it from other thoracic diseases. It provides clear visualization of the pleural surface, diaphragm, and mediastinal lymph nodes, revealing key features such as pleural thickening, nodularity, and mediastinal pleural involvement, which are critical for accurate diagnosis. Magnetic resonance imaging (MRI), though not commonly used for initial screening, offers superior accuracy in staging. It excels at detecting chest wall and diaphragmatic invasion, particularly when enhanced with specialized pulse sequences and gadolinium‐based contrast agents. MRI is especially useful for patients who are allergic to iodinated contrast media [29]. Positron emission tomography/CT (PET/CT) combines metabolic and anatomical imaging. It detects increased glucose uptake, which is typically higher in malignant mesothelioma than in benign pleural conditions, providing valuable information for preoperative staging [30]. Despite these advantages, no imaging modality offers adequate sensitivity for early‐stage diagnosis. CT‐guided pleural biopsy, though increasingly used, has a limited diagnostic yield of about 44%. In contrast, thoracoscopy remains the most accurate diagnostic method, with an accuracy rate exceeding 80% [31]. The TNM staging system, currently in its eighth edition as published by the International Association for the Study of Lung Cancer (IASLC) in 2016, remains the most widely adopted framework for clinical assessment of PM [32]. The Brigham staging system, developed at Brigham and Women's Hospital, places particular emphasis on tumor resectability and nodal involvement and is primarily employed to determine surgical candidacy for procedures such as pleurectomy/decortication (P/D) or extrapleural pneumonectomy (EPP) [33]. Historically, the Butchart system—first proposed in 1976—classified disease based on the anatomical extent of the primary tumor, largely through imaging‐based assessment. However, it has since been superseded by the more nuanced TNM classification in contemporary clinical practice (Table 1) [34].

**TABLE 1:** Comparison of Pleural mesothelioma (PM) staging systems.

Stage	TNM (IASLC, 8th edition)	Brigham staging system	Butchart staging system
Stage I	T1N0M0: Tumor limited to ipsilateral parietal and/or visceral pleura (± diaphragmatic or mediastinal pleura) without lymph node or distant metastasis	Tumor confined to ipsilateral pleura; no lymph node involvement; resectable	Tumor confined to ipsilateral pleura and possibly diaphragm
Stage II	T2N0–1M0: Tumor invades diaphragm or lung; may involve ipsilateral lymph nodes	Tumor with ipsilateral lymph node involvement; still resectable	Tumor invades chest wall, mediastinum, or regional lymph nodes
Stage III	T3N0–2M0 / T1–3N2M0: Locally advanced but potentially resectable; tumor may invade chest wall, pericardium, or mediastinal structures	Tumor invades vital mediastinal structures; may be unresectable	Tumor extends into peritoneum or contralateral pleura
Stage IV	T4 any N/any T N3/any M1: Unresectable disease; distant metastasis or extensive local invasion (e.g., spine, through diaphragm, peritoneum, contralateral lung)	Tumor with distant metastasis or unresectable invasion	Distant metastasis (e.g., liver, brain, bones)

Pathological reports must explicitly present the histological subtype and Ki‐67 proliferation index. In epithelioid PM, detailed descriptions encompassing growth pattern, nuclear grading, cellular atypia, mitotic figures, and necrosis extent are essential. Molecular diagnostics frequently employ biomarkers such as calretinin, CK5/6, WT‐1, mesothelin (MSLN), and D2‐40, whereas lung adenocarcinoma biomarkers typically include TTF‐1, napsin A, CEA, BerEP4, and claudin‐4 [35, 36]. For precise differential diagnosis, pathological evaluation should incorporate results from at least three biomarkers associated with both PM and lung adenocarcinoma. Additionally, BAP1 gene testing is advisable for younger patients lacking asbestos exposure history and familial tumor aggregation [37]. Importantly, CDKN2A mutations exhibit a strong association with poor prognosis, particularly prevalent in sarcomatoid mesothelioma [38]. Although neither BAP1 nor CDKN2A mutations provide absolute specificity in diagnosing malignant mesothelioma, they remain invaluable adjuncts for differentiating PM from benign pleural lesions and prognostic assessment. In recent years, advances in multi‐omics technologies have profoundly reshaped the molecular classification of PM. Blum and colleagues introduced a computational framework known as WISP (Weighted In Silico Pathology), which integrates transcriptomic and epigenetic profiles—including DNA methylation and microRNA (miRNA) expression—to quantitatively deconvolute the proportions of epithelioid and sarcomatoid components within tumor samples [39]. Notably, tumors exhibiting elevated S‐scores were enriched for aggressive molecular programs, including epithelial‐to‐mesenchymal transition, cell cycle progression, and TP53 signaling, and were associated with significantly poorer prognosis (hazard ratio = 6.28, *p* < 0.001). Intriguingly, the prognostic value of the S‐score surpassed that of standard histopathological subtypes. Analysis of 72 patients from the IFCT‐MAPS2 clinical trial revealed that a high transcriptomic S‐score, reflecting a greater sarcomatoid component, was significantly associated with prolonged progression‐free survival (PFS) in patients receiving combination therapy with nivolumab and ipilimumab [40]. In contrast, patients with low S‐scores derived no substantial benefit from the combination over monotherapy (Tables 2, 3, 4).

**TABLE 2:** Clinical trials related to PM therapy.

Title	Trial phase	Intervention	Number of participants	Result	Adverse effects	Reference number
Phase III study of pemetrexed in combination with cisplatin versus cisplatin alone in patients with malignant pleural mesothelioma	Phase 3	Pemetrexed + cisplatin vs. cisplatin alone	448	OS: 12.1 months (pemetrexed + cisplatin), 9.3 months (cisplatin alone); PFS: 5.7 months (pemetrexed + cisplatin), 3.9 months (cisplatin alone)	Fatigue, nausea, hematologic toxicities	[5]
Bevacizumab for newly diagnosed pleural mesothelioma in the Mesothelioma Avastin Cisplatin Pemetrexed Study (MAPS)	Phase 3	Bevacizumab + pemetrexed + cisplatin	448	OS: 18.8 months (bevacizumab group), 16.1 months (control group); PFS: 9.2 months (bevacizumab group), 6.0 months (control group)	Hypertension, proteinuria, bleeding events	[6]
First‐line nivolumab plus ipilimumab in unresectable malignant pleural mesothelioma (CheckMate 743)	Phase 3	Nivolumab + ipilimumab vs. standard chemotherapy	605	OS: 18.1 months (nivolumab + ipilimumab), 14.1 months (chemotherapy); PFS: 6.8 months (nivolumab + ipilimumab), 5.6 months (chemotherapy)	Fatigue, rash, diarrhea, colitis	[7]
Multicentre randomised phase III trial comparing pembrolizumab versus single‐agent chemotherapy for advanced pre‐treated malignant pleural mesothelioma: the PROMISE‐meso trial	Phase 3	Pembrolizumab vs. chemotherapy	195	OS: 13.3 months (pembrolizumab), 10.1 months (chemotherapy); PFS: 4.4 months (pembrolizumab), 4.6 months (chemotherapy)	Fatigue, rash, pneumonitis	[48]
A randomised phase III study of bevacizumab and carboplatin‐pemetrexed chemotherapy with or without atezolizumab as first‐line treatment for advanced pleural mesothelioma (BEAT‐meso trial)	Phase 3	Bevacizumab + carboplatin‐pemetrexed + atezolizumab	400	/	Not yet available	[61]
Nintedanib in combination with pemetrexed and cisplatin for chemotherapy‐naive patients with advanced malignant pleural mesothelioma (LUME‐Meso)	Phase 3	Nintedanib + pemetrexed + cisplatin vs. placebo	448	OS: 18.3 months (nintedanib group), 15.1 months (placebo group); PFS: 7.7 months (nintedanib group), 5.5 months (placebo group)	Diarrhea, fatigue, nausea, liver function abnormalities	[63]
Phase II study of cediranib in patients with malignant pleural mesothelioma: SWOG S0509	Phase 2	Cediranib + chemotherapy	45	OS: 7.3 months (cediranib group), 6.0 months (control group); PFS: 4.2 months (cediranib group), 3.5 months (control group)	Hypertension, fatigue, nausea, diarrhea	[68]
Phase II trial of cediranib in combination with cisplatin and pemetrexed in chemonaive patients with unresectable malignant pleural mesothelioma	Phase 2	Cediranib + pemetrexed + cisplatin	40	OS: 12.0 months (cediranib group), 9.5 months (control group); PFS: 6.8 months (cediranib group), 5.4 months (control group)	Hypertension, diarrhea, fatigue	[69]
Tremelimumab for patients with chemotherapy‐resistant advanced malignant mesothelioma: an open‐label, single‐arm, Phase 2 trial	Phase 2	Tremelimumab	25	mOS: 6.1 months; median PFS: 2.2 months	Fatigue, rash, and infusion‐related reactions	[94]
Tremelimumab as second‐line or third‐line treatment in relapsed malignant mesothelioma (DETERMINE): a multicentre, international, randomised, double‐blind, placebo‐controlled phase 2b trial	Phase 2b	Tremelimumab, placebo	571	OS: 12.1 months in the tremelimumab group vs. 9.3 months in placebo; PFS: 2.5 months vs. 2.6 months	Fatigue, liver function test abnormalities, and rash	[95]
Clinical safety and activity of pembrolizumab in patients with malignant pleural mesothelioma (KEYNOTE‐028): preliminary results from a non‐randomised, open‐label, phase 1b trial	Phase 1b	Pembrolizumab	25	OS: 18.0 months; PFS: 4.0 months	Fatigue, rash, and infusion‐related reactions	[96]
Clinical efficacy and safety of nivolumab: results of a multicenter, open‐label, single‐arm, Japanese phase II study in malignant pleural mesothelioma (MERIT)	Phase 2	Nivolumab	38	OS: 13.7 months; PFS: 3.3 months	Rash, diarrhea, and fatigue	[97]
Nivolumab versus placebo in patients with relapsed malignant mesothelioma (CONFIRM): a multicentre, double‐blind, randomised, phase 3 trial	Phase 3	Nivolumab, placebo	332	OS: 10.8 months in nivolumab group vs. 6.2 months in placebo group; PFS: 3.0 months vs. 2.0 months	Fatigue, rash, and gastrointestinal issues	[98]
Durvalumab with first‐line chemotherapy in previously untreated malignant pleural mesothelioma (DREAM): a multicentre, single‐arm, phase 2 trial with a safety run‐in	Phase 2	Durvalumab + first‐line chemotherapy	43	OS: 15.5 months; PFS: 6.2 months	Pneumonitis, fatigue, and infusion‐related reactions	[108]
Durvalumab with platinum‐pemetrexed for unresectable pleural mesothelioma: survival, genomic and immunologic analyses from the phase 2 PrE0505 trial	Phase 2	Durvalumab + platinum‐pemetrexed	65	OS: 18.7 months; PFS: 5.7 months	Pneumonitis, fatigue, and infusion‐related reactions	[109]
JME‐001 phase II trial of first‐line combination chemotherapy with cisplatin, pemetrexed, and nivolumab for unresectable malignant pleural mesothelioma	Phase 2	Cisplatin + pemetrexed + nivolumab	47	OS: 22.4 months; PFS: 6.1 months	Nausea, fatigue, and pneumonitis	[110]
Canadian Cancer Trials Group IND.227: a phase 2 randomized study of pembrolizumab in patients with advanced malignant pleural mesothelioma	Phase 2	Pembrolizumab	52	OS: 10.5 months; PFS: 3.5 months	Fatigue, rash, and gastrointestinal issues	[102]
Pembrolizumab plus chemotherapy versus chemotherapy in untreated advanced pleural mesothelioma in Canada, Italy, and France: a phase 3, open‐label, randomised controlled trial	Phase 3	Pembrolizumab + chemotherapy	450	OS: 18.2 months for combination group vs. 12.1 months for chemotherapy group; PFS: 7.0 months vs. 4.0 months	Fatigue, rash, and gastrointestinal issues	[103]
A phase I trial of regional mesothelin‐targeted CAR T‐cell therapy in patients with malignant pleural disease, in combination with the anti‐PD‐1 agent pembrolizumab	Phase 1	Mesothelin (MSLN)‐targeted CAR T‐cell therapy + pembrolizumab	/	OS and PFS not explicitly provided in summary. The combination showed encouraging anti‐tumor activity in PM	No severe adverse events reported. Mild‐to‐moderate effects from immunotherapy	[132]
EZH2 inhibitor tazemetostat in patients with relapsed or refractory, BAP1‐inactivated malignant pleural mesothelioma: a multicentre, open‐label, phase 2 study	Phase 2	EZH2 inhibitor (tazemetostat)	45	No improvement in OS or PFS, compared to standard treatment	Fatigue, nausea, and anemia were common. Some patients experienced moderate liver enzyme elevation	[150]
Vorinostat in patients with advanced malignant pleural mesothelioma who have progressed on previous chemotherapy (VANTAGE‐014)	Phase 3	Vorinostat (HDAC inhibitor)	660	mOS: 8.3 months in the vorinostat group vs 7.3 months in the placebo group (not statistically significant). mPFS: 3.2 months in vorinostat vs. 2.9 months in placebo	Nausea, fatigue, and gastrointestinal disturbances	[155]
A Phase II study of autologous dendritic cell vaccine for treatment of malignant pleural mesothelioma patients after chemotherapy	Phase 2	Autologous dendritic cell (DC) vaccine	40	No significant difference in OS or PFS between DC vaccine and chemotherapy alone	Mild adverse reactions: fever, fatigue, and local reactions at the injection site	[122]
Abemaciclib in patients with p16ink4A‐deficient mesothelioma (MiST2)	Phase 2	Abemaciclib	60	Abemaciclib showed an improvement in OS and PFS, compared to historical controls in patients with p16ink4A‐deficient mesothelioma. Median OS (mOS) was 18.7 months and PFS was 6.3 months	Common adverse effects included diarrhea, fatigue, nausea, and neutropenia	[191]
SWOG S0722: phase II study of mTOR inhibitor everolimus (RAD001) in advanced malignant pleural mesothelioma (PM)	Phase 2	Everolimus (RAD001)	80	Everolimus demonstrated limited efficacy in improving OS and PFS in patients with advanced PM. mOS was 12.5 months and PFS was 3.6 months	Most common adverse effects were fatigue, nausea, and mouth sores	[196]
Phase 1 cohort expansion study of LY3023414, a dual PI3K/mTOR inhibitor, in patients with advanced mesothelioma	Phase 1	LY3023414 (PI3K/mTOR inhibitor)	50	LY3023414 showed modest clinical activity with improvements in PFS in some patients. mOS was 14.2 months, while PFS was 4.5 months	Adverse effects included hyperglycemia, rash, and diarrhea	[197]
Phase I trial of hedgehog pathway inhibitor vismodegib (GDC‐0449) in patients with refractory, locally advanced or metastatic solid tumors	Phase 1	Vismodegib (GDC‐0449)	33	Vismodegib showed limited activity in mesothelioma, with mOS of 10.6 months and PFS of 2.3 months	Severe adverse effects included muscle spasms, alopecia, and dysgeusia	[211]
A phase I, multicenter, open‐label, first‐in‐human, dose‐escalation study of the oral smoothened inhibitor Sonidegib (LDE225) in patients with advanced solid tumors	Phase 1	Sonidegib (LDE225)	55	Sonidegib demonstrated minimal efficacy in mesothelioma, with mOS of 12.4 months and PFS of 3.2 months	Adverse effects included fatigue, diarrhea, and anorexia	[212]
Phase II clinical trial of amatuximab, a chimeric antimesothelin antibody with pemetrexed and cisplatin in advanced unresectable pleural mesothelioma	Phase 2	Amatuximab (anti‐MSLN antibody), pemetrexed, cisplatin	Not specified	mOS: 14.8 months. mPFS: 6.1 months	Nausea, fatigue, hematologic toxicity (e.g., anemia, neutropenia), liver enzyme elevation	[223]
First‐in‐human, multicenter, phase I dose‐escalation and expansion study of anti‐mesothelin antibody‐drug conjugate anetumab ravtansine in advanced or metastatic solid tumors	Phase 1	Anetumab ravtansine (AR; MSLN‐targeting antibody‐drug conjugate [ADC])	90	mOS was not reached at the time of reporting. mPFS: 3.9 months	Fatigue, nausea, liver toxicity, ocular toxicity (e.g., visual disturbances)	[227]
Safety and activity of anti‐mesothelin antibody‐drug conjugate anetumab ravtansine in combination with pegylated‐liposomal doxorubicin in platinum‐resistant ovarian cancer	Phase 1b	AR, pegylated‐liposomal doxorubicin	52	mOS was 15.7 months. PFS: Median PFS was 4.3 months	Hematologic toxicity, gastrointestinal effects (e.g., nausea), fatigue	[231]
First‐in‐human phase 1 study of ABBV‐085, an antibody‐drug conjugate (ADC) targeting LRRC15, in sarcomas and other advanced solid tumors	Phase 1	ABBV‐085 (LRRC15‐targeting ADC)	60	mOS was 9.7 months. Median PFS was 4.2 months	Fatigue, nausea, thrombocytopenia, neutropenia	[229]
Phase I study of lentiviral‐transduced chimeric antigen receptor‐modified T cells recognizing mesothelin in advanced solid cancers	Phase 1	Lentiviral‐transduced CAR T cells targeting MSLN	28	mOS was 7.3 months. PFS: Median PFS was 3.2 months	Cytokine release syndrome (CRS), neurotoxicity, transient fever, and fatigue	[227]
Phase I clinical safety and preliminary efficacy of PD‐1‐mesoCAR‐T cells in the treatment of malignant pleural/peritoneal mesothelioma	Phase 1	PD‐1‐mesoCAR‐T cells (chimeric antigen receptor T cells targeting MSLN)	18	mOS was not yet reached, with preliminary efficacy showing tumor response. PFS: Median PFS was 4.5 months	CRS, fatigue, fever, headache, gastrointestinal symptoms	[240]

**TABLE 3:** Epigenetic changes in PM.

Classification	Target	Mechanism	Effect and clinical application	Clinical trials
DNA methylation	FKBP5[150]	Methylation linked to PM risk and prognosis	Low methylation increases risk, poor prognosis	No
	MLLT1[150]	Methylation linked to PM risk and prognosis	High methylation increases risk, poor prognosis	No
	RAP1A [151]	Differential methylation associated with early diagnosis	Early diagnostic marker	No
	LHX6 [151]	Differential methylation associated with early diagnosis	Early diagnostic marker	No
	HOOK2 [151]	Differential methylation associated with early diagnosis	Early diagnostic marker	No
	APC [152]	Hypomethylated in PM	Tumor suppressor loss	No
	CDH1 [152]	Hypermethylated in PM	Impaired cell adhesion	No
	ESR1 [152]	Hypermethylated in PM	Estrogen receptor silencing	No
	miR‐34b/c [152]	Hypermethylated in PM	Tumor suppressor silencing	No
	PGR [152]	Hypermethylated in PM	Progesterone receptor silencing	No
	RARβ [152]	Hypermethylated in PM	Retinoic acid receptor silencing	No
	SFRP1 [152]	Hypermethylated in PM	Wnt pathway inhibition	No
	WIF1 [152]	Hypermethylated in PM	Wnt pathway inhibition	No
	UHRF1 [159]	Recruits DNA methyltransferases, regulates DNA methylation and chromatin	Inhibits proliferation, invasion	No
Histone modifications	KDM4A [153]	Histone demethylase, opens chromatin for transcription	Suppresses growth and enhances apoptosis	No
	LSD1 [155]	Demethylates histones, induces epithelial transition	Enhances cisplatin sensitivity, promotes epithelial shift	No
	EZH2 [162]	Catalytic subunit of PRC2, silences genes by methylating H3K27	Restores tumor suppressor gene activity, inhibits tumor growth	Yes (tazemetostat)
	HDACs [167]	Removes acetyl groups from histones, represses gene transcription	Reduces tumor growth, enhances cisplatin sensitivity	Yes (panobinostat, vorinostat)

**TABLE 4:** Dysregulated non‐coding RNAs with biological activity in PM.

RNA type	Name	Experimentally validated function(s)	Clinical application	Clinical trials
miRNA	miR‐16‐5p [176]	Inhibits apoptosis‐related genes, promotes apoptosis	Chemotherapy resistance, PD‐L1 modulation	Yes
miRNA	miR‐193a‐3p [177]	Inhibits PM tumor growth	Potential anti‐tumor effect	No
miRNA	miR‐137‐3p [174]	Inhibits PM tumor growth	Potential anti‐tumor effect	No
miRNA	miR‐34b/c [174]	Inhibits PM cell proliferation, promotes apoptosis	Enhances chemotherapy	No
miRNA	miR‐215‐5p [178]	Inhibits PM tumor growth	Potential anti‐tumor effect	No
miRNA	miR‐34a‐5p [179]	Induces G0/G1 arrest, enhances cisplatin effects	Enhances cisplatin efficacy	No
miRNA	miR‐126 [180]	Inhibits angiogenesis, induces apoptosis	Delivered via exosomes	No
miRNA	miR‐206 [181]	Inhibits CDK6, reduces tumor growth	Combined with CDK4/6 inhibitor	No
miRNA	miR‐106b [182]	Suppresses migration and invasion	Potential metastasis inhibitor	No
miRNA	miR‐142‐3p [183]	Inhibits integrin αV, reduces adhesion and invasion	Potential anti‐tumor effect	No
miRNA	miR‐182 [185]	Promotes PM cell proliferation, invasion	Target for antisense oligonucleotides	No
miRNA	miR‐183 [185]	Promotes PM cell proliferation, invasion	Target for antisense oligonucleotides	No
lncRNA	PVT1 [190]	Upregulates FOXM1, promotes cell proliferation	Potential therapeutic target	No
lncRNA	LINC00152 [191]	Regulates proliferation and migration via EZH2 interaction	Basis for combination therapy	No
lncRNA	Linc00941 [192]	Promotes c‐MYC protein synthesis, enhances gene expression	Potential therapeutic target	No

## 4 Treatment and Prognosis

### 4.1 Surgical Intervention

Surgery remains one of the cornerstone treatments for patients with PM, particularly those with localized disease. The primary surgical approaches include P/D and EPP [41, 42]. P/D involves the removal of the affected pleura and any visible tumor masses, while EPP is a more extensive procedure that entails resection of the ipsilateral pleura, lung, diaphragm, and part of the pericardium. Several studies have demonstrated that P/D is associated with a significantly lower perioperative mortality rate and better long‐term survival, compared to EPP. For instance, a retrospective analysis revealed that the 30‐day and 90‐day mortality rates for EPP were 8.0% and 14.1%, respectively, while those for P/D were 5.4% and 12.8%, respectively [43]. A systematic review and meta‐analysis further corroborated these findings, showing that P/D offers superior OS and fewer postoperative complications than EPP [42]. Research has also indicated that the complication rates for EPP are higher than for P/D, particularly concerning heart failure and pneumonia, whereas the primary complication associated with P/D is postoperative air leakage [44].

It is noteworthy that a study comparing the quality of life in patients undergoing EPP versus P/D found no significant differences between the two groups postoperatively, although a marked decline in lung function was observed in the EPP cohort [45]. During long‐term follow‐up, P/D has shown more favorable PFS and post‐recurrence survival. One study reported a median PFS of 21 months for P/D, compared to 13 months for EPP [46]. Overall, P/D appears to be superior to EPP in terms of reducing perioperative mortality, improving long‐term survival, and minimizing postoperative complications. P/D should be considered the preferred surgical option for patients with PM, except in cases where specific clinical conditions necessitate a more aggressive EPP approach.

### 4.2 Chemotherapy

Conventional chemotherapy regimens, primarily based on platinum compounds (such as cisplatin or carboplatin) combined with pemetrexed, have long been the cornerstone of PM treatment, although the OS benefit has been modest [47]. The efficacy of monotherapy is generally limited; for example, gemcitabine or vinorelbine alone has shown low objective response rates in recurrent PM [48]. The rapid emergence of chemotherapy resistance following initial treatment is the primary factor affecting the efficacy of PM therapy. Research into the mechanisms underlying chemoresistance in PM remains inadequate. One study suggests that aquaporin‐6 (AQP‐6), localized to the plasma membrane and certain intracellular structures, enhances cellular resistance to oxidative stress by promoting the efflux of hydrogen peroxide, thereby contributing to chemoresistance [49]. Another study found that in cisplatin‐responsive PM cell lines, phosphorylation levels of AKT1 and GSK3B were reduced, while those of JNK1/2/3 were increased. In contrast, in cisplatin‐resistant cell lines, phosphorylation of AKT1 and GSK3B was unaffected, and JNK1/2/3 phosphorylation was reduced, indicating that kinase phosphorylation and activity may play critical roles in cellular responses to chemotherapy [50]. Moreover, genetic mutations and epigenetic alterations are crucial in driving chemoresistance in PM. Mutations or deletions in genes such as BAP1, NF2, and CDKN2A significantly affect tumor cell responsiveness to chemotherapeutic agents, resulting in higher resistance and poorer prognosis [51]. Novel chemotherapy approaches, such as intrapleural cisplatin‐fibrin chemotherapy, have shown promise in achieving better tumor control with reduced systemic toxicity [52]. It seems that maintaining high local drug concentrations is an effective approach to overcome chemoresistance. Eosinophils impair cisplatin‐pemetrexed‐induced apoptosis in mesothelioma via secretion of Charcot‐Leyden crystal protein (CLC‐P/Galectin‐10). Targeting CLC‐P or depleting eosinophils restores chemosensitivity, indicating potential for therapeutic intervention in eosinophil‐driven treatment resistance [53].

Hyperthermic intrathoracic chemotherapy (HITOC) is a regional therapeutic approach that involves the intrapleural administration of cisplatin‐based chemotherapy at fever‐range temperatures (40°C–43°C) following cytoreductive surgery. Systematic evidence suggests that HITOC may prolong survival and delay disease progression, particularly when administered at higher doses. Despite some associated toxicities, the treatment is generally well tolerated, supporting its further evaluation as a perioperative therapeutic strategy [54]. Beyond its direct cytotoxic effect, emerging evidence suggests that HITOC profoundly remodels the tumor immune microenvironment. It enhances T‐cell infiltration, promotes cytotoxic immune responses, and upregulates immune checkpoint molecule expression, thereby sensitizing tumors to immune checkpoint blockade. In preclinical mesothelioma models, HITOC synergized with dual PD‐1 and CTLA‐4 inhibition to improve survival outcomes, supporting its potential as an immunomodulatory adjunct to immunotherapy in future clinical applications [55].

In conclusion, although chemotherapy remains a key component in the treatment of PM, its efficacy is limited and accompanied by substantial toxicity. This underscores the importance of developing novel therapeutic strategies.

### 4.3 Anti‐Angiogenic Therapy

Up to now, numerous attempts have been made in the realm of anti‐angiogenic therapy for PM, with the most prominent being the anti‐VEGF agent, bevacizumab. Other agents include certain tyrosine kinase inhibitors and small molecular compounds. However, research in the field of anti‐angiogenic therapy for PM remains relatively insufficient, and the outcomes so far have been decidedly mixed.

Several early clinical trials involving bevacizumab have suggested that it may not significantly improve PFS or OS in patients with PM and is accompanied by a relatively high incidence of adverse events (AEs) [56, 57, 58, 59]. However, in the MAPS trial, a Phase II/III, multicenter, open‐label, randomized clinical study aimed at evaluating the effect of bevacizumab on survival in patients with diffuse PM, bevacizumab in combination with standard chemotherapy (pemetrexed/cisplatin) significantly improved both PFS and OS, compared to chemotherapy alone. Among 449 patients, the median PFS was 9.2 months in the bevacizumab group, compared to 7.3 months in the control group, and the median OS (mOS) was 18.8 months, compared to 16.1 months in the control group, both demonstrating substantial survival benefits (PFS: HR = 0.61, OS: HR = 0.75). Despite these promising results, the bevacizumab group exhibited higher toxicity, with an increased incidence of AEs, particularly hypertension, cardiovascular events, and thrombotic complications, although most of these adverse reactions were manageable and did not necessitate treatment discontinuation. Furthermore, long‐term follow‐up indicated that the addition of bevacizumab to standard chemotherapy in advanced PM patients did not negatively impact health‐related quality of life and even seemed to alleviate peripheral neuropathy and pain to some extent [60]. The latest ETOP 13–18 BEAT‐Meso trial, an international, multicenter, open‐label, 1:1 randomized Phase III study, aimed to assess the therapeutic effect of atezolizumab combined with bevacizumab and standard chemotherapy (carboplatin and pemetrexed) in patients with diffuse PM [61]. In the experimental arm, patients received carboplatin (AUC 5) + pemetrexed (500 mg/m^2^ every 3 weeks for 4–6 cycles) in combination with bevacizumab (15 mg/kg every 3 weeks until disease progression or intolerable toxicity) and atezolizumab (1200 mg every 3 weeks until disease progression or intolerable toxicity). The control group received standard chemotherapy (carboplatin + pemetrexed) along with bevacizumab. The combination therapy demonstrated a significant advantage in PFS (9.2 months vs. 7.6 months, *p* = 0.0021), although no statistically significant difference in OS was observed (18.8 months vs. 16.1 months, *p* = 0.14). Subgroup analysis revealed that in non‐epithelioid patients, the combination therapy significantly outperformed the control group in both OS and PFS. The ASCO and NCCN guidelines suggest that, in the absence of cardiovascular contraindications to bevacizumab, the combination of bevacizumab, pemetrexed, and platinum‐based chemotherapy may be considered as a first‐line treatment for patients with unresectable, PS 0–2 mesothelioma [4, 62]. However, further studies are warranted to enhance its efficacy and mitigate its cardiovascular‐related adverse effects.

Besides bevacizumab, researchers have also attempted to incorporate tyrosine kinase inhibitors, such as nintedanib, into the treatment of PM. However, the therapeutic outcomes have been less than satisfactory. The Phase III LUME‐Meso trial [63], designed to evaluate the efficacy of nintedanib in combination with carboplatin/pemetrexed in patients with unresectable PM, demonstrated no significant difference in PFS or OS between the nintedanib and placebo groups. The median PFS was 6.8 months in the nintedanib group, compared to 7.0 months in the placebo group (HR = 1.01, 95% CI: 0.79–1.30, p = 0.91), and the mOS was 14.4 months in the nintedanib group versus 16.1 months in the placebo group (HR = 1.12, 95% CI: 0.79–1.58, *p* = 0.538). A recent Phase II clinical trial evaluating nintedanib in patients with relapsed PM revealed a median PFS of 1.8 months (95% CI: 1.68, 3.55), with a 4‐month PFS rate of 13%. The mOS was 4.2 months (95% CI: 2.53, 8.74), with a 4‐month OS rate of 55% [64]. Overall, the monotherapy with nintedanib failed to meet expectations. In another study, a combination of nintedanib and pembrolizumab showed some degree of efficacy, with a disease control rate (DCR, including partial response and stable disease) of 46.6% at 6 months, yet the results were still far from promising [65]. Further investigations revealed that the resistance of PM patients to the combination of anti‐PD‐1 and anti‐angiogenic therapy was associated with an increase in tumor‐infiltrating regulatory T cells (Tregs), elevated systemic pro‐inflammatory cytokines (such as IL‐6, CXCL8, and VEGF), and genomic instability, including chromosomal 9 deletions and high somatic copy number variations [66]. Another VEGF‐R tyrosine kinase inhibitor, celecoxib, was tested in several clinical trials, but its monotherapy efficacy was limited and accompanied by notable toxicity. Although the combination with chemotherapy demonstrated some preliminary efficacy, the associated toxicities hindered its clinical applicability, thereby preventing further investigation [67, 68, 69, 70].

### 4.4 Immunotherapy

#### 4.4.1 Immune Microenvironment

**FIGURE 3:**
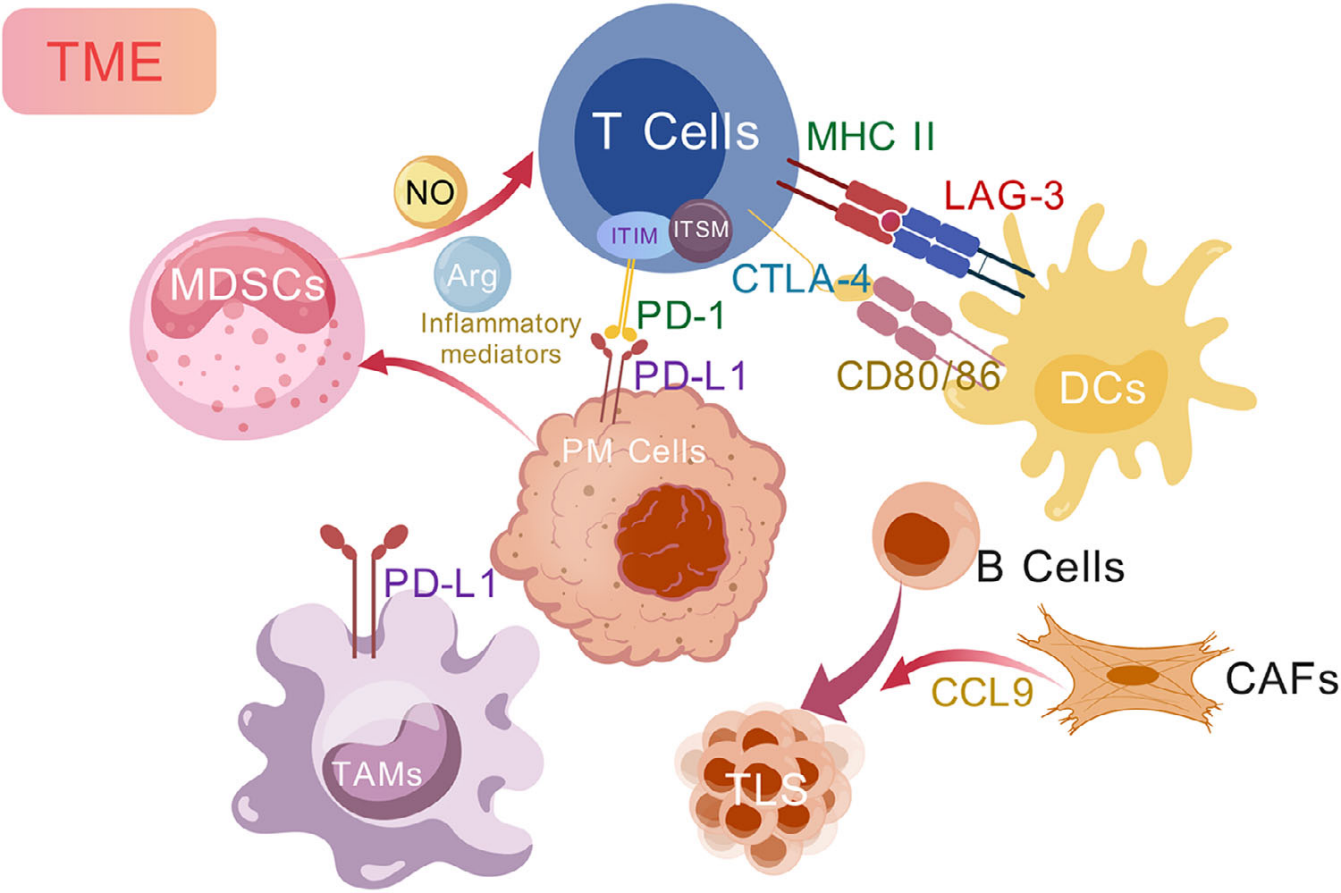
Schematic representation of the tumor microenvironment in malignant PM. MDSCs: myeloid‐derived suppressor cells; DCs: dendritic cells; TAMs: tumor‐associated macrophages; TLS: tertiary lymphoid structures.

Immunotherapy harnesses the adaptive immune system to target and eliminate cancer cells. Therefore, cytotoxic immune cells must infiltrate the tumor microenvironment (TME). Increasing evidence supports the notion that PM is an “immune‐cold” tumor, characterized by a low median tumor mutation burden (TMB) and a paucity of immune cell infiltration within its TME [23, 71]. Moreover, tumor‐infiltrating lymphocytes (TILs) in PM are often dominated by immunosuppressive cells, such as tumor‐associated macrophages (TAMs) and myeloid‐derived suppressor cells (MDSCs), with a relative paucity of tumor‐suppressive cells like cytotoxic T lymphocytes (CTL), B cells, and NK cells [72, 73] (Figure 3). Single‐cell RNA sequencing identified four cancer‐intrinsic programs associated with poor prognosis in PM: cell cycle, epithelial–mesenchymal transition (EMT), oxidative stress response, and neuroendocrine‐like phenotype [74]. These programs are linked to proliferation, invasiveness, and immune evasion. Tumors with sarcomatoid‐like signatures were associated with an altered immune microenvironment enriched in CXCL9⁺ macrophages, fetal‐like endothelial cells, and diverse T‐cell subsets.

TAMs constitute a substantial proportion (20%–50%) of all TILs and frequently express high levels of programmed death‐ligand 1 (PD‐L1) [75]. In PM, TAMs effectively inhibit the function of CTL [76, 77]. Specific biomarkers, such as CD68 and CD163, used to identify TAMs, have shown that the presence of immunosuppressive macrophages correlates with poor prognosis in patients with epithelioid PM [72, 78]. Immunosuppressive macrophages play a central role in the TME of PM, largely driven by CSF‐1R signaling through CSF‐1 and IL‐34. To simultaneously neutralize CSF‐1 and IL‐34‐mediated immunosuppressive signaling, an engineered high‐affinity decoy receptor fusion protein has been developed [79]. This molecule effectively blocks CSF‐1R activation by capturing both ligands, thereby suppressing the differentiation of tumor‐induced immunosuppressive macrophages and enhancing CD8⁺ T‐cell‐mediated cytotoxicity in preclinical models. This approach offers a novel strategy to modulate the tumor immune microenvironment.

MDSCs are a heterogeneous population of immature myeloid cells that typically increase as the tumor progresses and are often identified by immunohistochemical markers like CD15+, CD11b+, and CD66b+ [80]. Tumor and host cells produce various pro‐inflammatory mediators that activate MDSCs, driving their accumulation and suppressive activity. MDSCs inhibit T‐cell activation, induce other immunosuppressive cells, regulate inflammation in the TME, and facilitate a shift of the immune response toward a pro‐tumorigenic state [81]. Through the production of nitric oxide (NO) and arginase, MDSCs suppress T‐cell proliferation and function, thereby promoting immune evasion by the tumor [82]. Studies have confirmed a marked increase in MDSC levels in PM patients, including their detection in pleural effusions, with high MDSC expression correlating with poorer prognosis [83]. Additionally, research has shown that the COX‐2 inhibitor celecoxib can reduce MDSC numbers in murine models of mesothelioma and enhance the efficacy of immunotherapy [84].

The immune cell infiltrate in mesothelioma includes 20%–40% T cells, predominantly CD8+ T cells, with a notable proportion expressing exhaustion markers such as TIM‐3 (39%), LAG‐3 (26%), and PD‐1 (32%) [85, 86]. Additionally, the relative proportions of different T‐cell subsets within the immune microenvironment are critical prognostic factors. Studies have indicated that tumors with low infiltration of T‐helper 2 cells and high infiltration of cytotoxic T cells exhibit upregulated immune and inflammation‐related pathways, suggesting that these patients may have a higher likelihood of responding to immune checkpoint inhibitors (ICIs) [87]. A transcriptomic study identified key genes linked to T‐cell exclusion, including SOX4, KDM5B, and several druggable targets such as SMO and ERBB2 [88]. Notably, high SMO expression correlated with poor response to nivolumab plus ipilimumab, implicating Hedgehog signaling as a potential target to overcome immune resistance.

Approximately 50% of PM patient samples exhibit high levels of B‐cell infiltration, which generally correlates with a better prognosis [89, 90]. Furthermore, studies have found that B‐cell infiltration and the formation of tertiary lymphoid structures (TLS) are more common in long‐term survivors with epithelioid PM, suggesting a potentially crucial role for B cells in modulating the aggressiveness of the disease [91]. Cancer‐associated fibroblasts (CAFs) are commonly recognized in solid tumors for their role in fostering an immunosuppressive TME through the secretion of various cytokines and growth factors [92]. However, recent findings in colorectal cancer liver metastases suggest that CAFs may promote TLS formation by secreting CCL19 to recruit B cells [93]. Nevertheless, the role and mechanisms of TLS formation in PM patients remain to be fully elucidated.

#### 4.4.2 ICIs

ICIs targeting CTLA‐4 and PD‐1/PD‐L1 are the primary immunotherapies for PM. The anti‐CTLA‐4 monoclonal antibody tremelimumab was one of the first to enter clinical trials. Its efficacy was initially assessed in the Phase II MESOT‐TREM 2008 trial, which included patients with PM or peritoneal mesothelioma who had relapsed after first‐line platinum‐based chemotherapy. Some patients demonstrated anti‐tumor activity, with an objective response rate (ORR) of 6.9%, and an mOS of 10.7 months [94]. However, regrettably, in larger‐scale clinical trials, tremelimumab failed to significantly improve OS. The mOS was 7.7 months in the tremelimumab group, compared to 7.3 months in the placebo group (HR = 0.92; *p* = 0.41) [95]. These studies suggest that while anti‐CTLA‐4 therapy holds some promise for the treatment of PM, further research is required to optimize therapeutic strategies.

In contrast, PD‐1/PD‐L1 inhibitors have shown more promise. Following the success of anti‐PD‐1/PD‐L1 immune therapies in other solid tumors, clinical investigations into anti‐PD‐1/PD‐L1 treatment for PM have gained significant momentum. Among these, the KEYNOTE‐28 trial [96], published in 2017, explored the safety and efficacy of pembrolizumab in PD‐L1‐positive patients with PM, reporting an ORR of 20%, a median PFS of 5.5 months, and an mOS of 18.7 months, with higher response rates observed in PD‐L1‐high expressing patients. Subsequently, the MERIT study from Japan affirmed the efficacy of nivolumab in PM, demonstrating objective therapeutic responses in some patients, a DCR of 68%, and an mOS of 17.3 months [97]. Notably, both studies revealed that patients with high PD‐L1 expression exhibited a more favorable response to ICIs. This was followed by two Phase III clinical trials. The PROMISE‐meso trial, the first randomized study assessing the efficacy of pembrolizumab in PM patients who had progressed after prior platinum‐based chemotherapy, unfortunately yielded negative results. Despite showing improvement in ORR, compared to single‐agent chemotherapy, neither PFS nor OS was significantly improved [48]. In contrast, the CONFIRM trial, a Phase III study evaluating nivolumab, found an mOS of 9.2 months in the nivolumab group, compared to 6.6 months in the placebo group (HR = 0.72; 95% CI: 0.55–0.94; *p* = 0.018) [98]. These clinical trials highlight the potential of PD‐1/PD‐L1 immune therapies, although further investigation is necessary to fully refine and optimize their application.

Given the success of combined CTLA‐4 and PD‐1/PD‐L1 therapies in other solid tumors, combination immunotherapy has become a promising approach for PM [99, 100]. The NIBIT‐MESO‐1 Phase II trial demonstrated that tremelimumab and durvalumab improved mOS to 16.6 months, with higher survival rates in patients with high TMB [101, 102]. Subsequent Phase II trials like MAPS‐2 and INITIATE also confirmed the efficacy of combination immunotherapy [103, 104]. The Phase III CM‐743 trial showed that nivolumab plus ipilimumab significantly outperformed chemotherapy, with a median survival of 18.1 months versus 14.1 months, leading to FDA approval of this combination as the first new first‐line PM therapy since 2004 [7, 105].

Future research is focused on combining chemotherapy with immunotherapy, as chemotherapy has been shown to enhance the immune response against tumors [106, 107]. Phase II trials such as DREAM, PrE0505, JME‐001, and IND‐227 have all suggested the potential benefits of combining immunotherapy with chemotherapy, showing improved outcomes in PM patients [108, 109, 110, 111]. A Phase III trial further validated pembrolizumab plus chemotherapy in extending OS, compared to chemotherapy alone, despite a slight increase in AEs [112]. The Phase III BEAT‐meso trial evaluated the efficacy of an ABC regimen—comprising atezolizumab, bevacizumab, and chemotherapy—as first‐line treatment for PM. Although no statistically significant improvement in OS was observed in the intention‐to‐treat population (20.5 vs. 18.1 months; *p* = 0.14), the combination therapy conferred a marked survival benefit in the non‐epithelioid PM subgroup, significantly prolonging both OS (17.9 vs. 10.0 months; HR = 0.50) and PFS (7.0 vs. 5.6 months; HR = 0.43). Notably, this regimen also demonstrated potential clinical benefit among PD‐L1‐positive patients and other subgroups with poor prognostic profiles. Despite a higher treatment discontinuation rate, the ABC regimen did not significantly compromise patient‐reported quality of life [61].

It is generally believed that high PD‐L1 expression is associated with poorer prognosis, although its predictive value in ICIs requires further study [113, 114]. Several recent Phase III trials do not support PD‐L1 as a reliable biomarker for predicting ICI benefits in PM [115]. Interestingly, PD‐1 expression within the PM immune microenvironment may serve as an independent predictor of response to ICIs, with patients exhibiting PD‐1+ CD8+ T cells and PD‐1+ CD68+ macrophages deriving greater benefit from ICI therapy [116]. Recent studies suggest that CD4+ T cells must cluster with CD8+ T cells on the same dendritic cell (DC) to form a tri‐cellular complex, which could partially predict patient responses to ICI treatment by quantifying these clusters [117]. A recent study developed transcriptomic T‐ and B‐cell signatures to quantify immune infiltration [118]. In ICI‐naïve MPM cohorts, high B‐cell infiltration was significantly associated with improved OS, supporting its prognostic relevance. In contrast, among patients treated with second‐line nivolumab plus ipilimumab, only T‐cell infiltration was predictive of objective response and survival benefit (hazard ratio = 0.06), whereas B‐cell infiltration lacked predictive value. These findings suggest that T‐cell infiltration serves as a specific predictor of benefit from dual ICI therapy, while B‐cell infiltration is primarily a prognostic marker in ICI‐naïve disease.

Additionally, ICI efficacy may be influenced by specific genomic characteristics. Extensive chromosomal rearrangements can facilitate neoantigen generation, thereby promoting T‐cell activation and enhancing responsiveness to ICIs [119, 120]. However, the deletion of the 9p21 region, involving CDKN2A and MTAP genes, is associated with decreased immune cell infiltration and lower PD‐L1 levels, potentially leading to resistance to ICI therapy [121, 122, 123]. Furthermore, BAP1 inactivation, along with higher LAG3 and VISTA gene expression, may enhance PM responsiveness to ICIs [124]. Nevertheless, BAP1 mutations alone cannot reliably serve as predictive biomarkers for ICI efficacy in PM patients, and the precise mechanisms by which BAP1 influences ICI response remain to be fully elucidated and warrant further investigation [125]. A recent study characterized pleural and peritoneal mesotheliomas by integrating exome and transcriptomic sequencing with multiplex immunofluorescence, focusing on BAP1, CDKN2A, MTAP, and NF2 status [126]. Tumors were classified into four immune microenvironment subtypes: fibrotic, immune desert, immune‐enriched fibrotic, and immune‐enriched non‐fibrotic. Loss of MTAP or CDKN2A was associated with globally reduced immune infiltration and predominance of the immune desert phenotype, whereas NF2 alterations correlated with increased infiltration of regulatory and total T lymphocytes. Although immune cell abundance did not directly predict immunotherapy outcomes, patients with BAP1 alterations experienced improved survival on nivolumab plus ipilimumab, while those with CDKN2A or NF2 mutations had poorer outcomes.

Dickkopf‐3 enhances the efficacy of programmed cell death protein 1 blockade by activating the p53 pathway, upregulating PD‐L1, and suppressing colony‐stimulating factor 1, thereby reshaping the tumor immune microenvironment. In a Phase II clinical trial, intratumoral adenovirus‐mediated Dickkopf‐3 combined with nivolumab demonstrated acceptable safety and preliminary efficacy in chemotherapy‐refractory epithelioid PM, yielding a 16.6% objective response rate and an mOS of 14.5 months [127]. A Phase I clinical trial (AdvanTG‐105) has observed partial responses in two epithelioid patients treated with a combination of anti‐TIGIT and anti‐PD‐1 antibodies, highlighting TIGIT as a promising immunotherapeutic target for this histological subtype [128]. Co‐inhibition of TIGIT and PD‐1 demonstrated superior efficacy in mesothelioma models. In vivo, this combination achieved a 90% objective response rate, outperforming anti‐PD‐1 monotherapy, PD‐1 plus CTLA‐4, and chemotherapy [129]. The regimen enhanced CD8⁺ T‐cell and NK‐cell activity, suppressed tumor growth, and showed favorable safety. Notably, treated animals remained tumor‐free upon rechallenge, suggesting durable immune memory and clinical potential.

#### 4.4.3 Is ICI Necessary?

It is important to note that despite the apparent promise of ICIs as a preferred treatment option for PM, recent studies suggest that the use of ICIs in PM patients warrants careful consideration. Literature has highlighted several issues with the biological rationale for ICI treatment in mesothelioma, as well as design flaws in clinical trials. These include the complexities of immunobiology, statistical limitations, informative censoring, and challenges related to clinical equipoise [130]. The previously mentioned IDN22773 trial [112] is a Phase III clinical study aimed at comparing the efficacy and safety of adding pembrolizumab to standard platinum‐based chemotherapy (cisplatin and pemetrexed). The primary endpoint of the trial was OS, and the results indicated that the addition of pembrolizumab to chemotherapy led to a modest improvement in OS. The mOS in the chemotherapy group was 16.1 months, while the immunotherapy group achieved 17.3 months. Although a statistically significant difference was observed, the difference between the two groups was minimal. Furthermore, it is essential to recognize that clinical trials often involve strictly selected patient cohorts, which may not fully reflect the therapeutic outcomes observed in real‐world settings. Recent real‐world data studies on ICI treatment for mesothelioma have yielded results that diverge from those seen in clinical trials. The Australian RIOMeso study [131], which included 119 patients with PM, found that 75% of the patients received a dual ICI regimen (ipilimumab and nivolumab) as first‐line therapy. The mOS for patients receiving first‐line treatment was 14.5 months, with no significant survival benefit, compared to the chemotherapy groups in CheckMate‐743 (14.1 months) and IND227 (16.1 months) [131]. A similar outcome was observed in a Dutch study analyzing 184 patients treated with nivolumab and ipilimumab, with the resulting mOS of 14.1 months showing no distinct advantage over previous clinical trials [132]. Additionally, a retrospective cohort study reviewing the treatment records of 90 PM patients found that the 12‐month OS for patients receiving immunotherapy, chemotherapy, and best supportive care (BSC) was 72%, 64%, and 29%, respectively, with no significant survival difference between chemotherapy and immunotherapy [133].

In the 3‐year follow‐up analysis of the CM‐743 trial, investigators performed stratified analyses based on histological subtypes. Patients with non‐epithelioid PM demonstrated a pronounced survival advantage when treated with the combination of ipilimumab and nivolumab, compared with standard chemotherapy (cisplatin plus pemetrexed). However, it is noteworthy that the CM‐743 trial was not originally powered or specifically stratified for this histological subgroup. The relatively small sample size in the non‐epithelioid cohort brings concerns of being statistically underpowered, raising the possibility of effect size inflation or false‐positive findings [134]. Therefore, while the observed outcomes are promising, current evidence remains insufficient to warrant an immediate shift in clinical practice. A separate study using statistical reconstruction and cost‐effectiveness modeling raised further concerns regarding the robustness and generalizability of CM‐743 trial outcomes. The survival‐inferred fragility index (SIFI) across endpoints was strikingly low (< 1%), indicating that outcome significance could be overturned by the random reassignment of as little as 1% of participants, highlighting the potential fragility of the reported benefit. In contrast, the MAPS trial lost statistical significance even prior to SIFI computation, with a negative SIFI value, suggesting an extreme sensitivity of results to statistical assumptions [135]. The Phase III BEAT‐meso trial reported that the ABC regimen (atezolizumab, bevacizumab, and chemotherapy) significantly improved both OS and PFS in patients with non‐epithelioid PM, supporting its potential role as a first‐line therapeutic strategy in this high‐risk subgroup. Conversely, in epithelioid PM, although a trend toward improved PFS was observed, no OS benefit was demonstrated, casting doubt on its clinical utility in this histological subset47 [61]. Collectively, these findings underscore the necessity of histology‐tailored immunotherapeutic strategies and highlight the need for further studies to delineate the optimal patient populations and treatment contexts for ABC‐based regimens.

Another important consideration regarding dual immunotherapy is the associated risk of toxic side effects. Several clinical trials have reported AEs induced by ICIs. In the JME‐011 Phase II trial, Miyamoto and colleagues observed that 55.6% of PM patients treated with nivolumab experienced Grade 3 or higher AEs, suggesting that ICIs may provoke significant toxic reactions [110]. The MAPS2 trial demonstrated that while no Grade 5 AEs occurred in the nivolumab monotherapy group, 5% of patients in the combination therapy arm succumbed to Grade 5 AEs [103]. In the CM‐743 trial, the combination of nivolumab and ipilimumab resulted in a 21% incidence of any grade severe AEs, markedly higher than the 8% observed in the chemotherapy group [7]. In the IND227 trial, the incidence of Grades 3–4 AEs in the pembrolizumab plus chemotherapy group was 27%, substantially higher than the 15% observed in the chemotherapy monotherapy group. Moreover, the hospitalization rate (18%) and treatment discontinuation rate (37%) were notably higher in the pembrolizumab group, compared to the chemotherapy group (6% and 20%, respectively) [112]. While immunotherapy demonstrates potential in the treatment of PM, the high incidence of severe AEs, particularly in combination therapy, underscores the possibility of increased treatment‐related toxicity. Therefore, a careful evaluation of the risk–benefit ratio is essential when considering its clinical application.

ICIs have demonstrated notable efficacy in PM, particularly with the combination of nivolumab and ipilimumab, which has shown superior OS in certain clinical trials, compared to traditional chemotherapy. However, the existing data remain insufficient, necessitating further real‐world evidence to substantiate their effectiveness. Additionally, the side effects associated with immunotherapy should not be overlooked, especially the relatively high incidence of Grade 3 and higher AEs. Therefore, while ICIs offer a promising therapeutic option for PM patients, vigilant monitoring of adverse effects is crucial, alongside heightened awareness of potential severe toxicities. Future research should focus on optimizing treatment regimens, minimizing toxicity, and further elucidating the efficacy of immunotherapy across different patient subgroups.

## 5 Prospective Advances in the Management of PM

### 5.1 Advancing Immunotherapy

#### 5.1.1 DC Vaccine Therapy

DC vaccine therapy involves extracting DCs from a patient's body, exposing them ex vivo to cancer cells or antigens, and then reintroducing them into the patient to activate an immune response against PM. Since 2004, studies have demonstrated that this approach can induce CTL responses against PM in vitro and inhibit tumor growth and enhance antitumor immunity in animal models. Several early clinical trials have further shown that DC vaccines can significantly improve survival in PM patients. For example, a Phase I clinical trial (NCT00280982) reported an mOS of approximately 19 months for patients receiving DC vaccine therapy [136, 137, 138].

The recent DENIM study, an open‐label, multicenter, randomized Phase II/III trial, randomly assigned patients to receive DC therapy plus BSC or BSC alone following first‐line chemotherapy, according to the local investigator's assessment [139]. The latest data from this trial showed an mOS of 16.9 months for patients receiving the DC vaccine combined with BSC, compared to 18.3 months for those receiving BSC alone. Although there was no significant improvement in PFS, the researchers speculate that the lack of OS benefit may be attributed to the prolonged interval between the end of chemotherapy and the initiation of DC vaccination, during which disease progression occurred in many patients. Current investigations are focused on the potential use of this vaccine earlier in the disease course or in combination with ICIs [140]. These findings suggest that DC vaccines represent a promising immunotherapeutic strategy for PM treatment.

#### 5.1.2 CAR T‐Cell Therapy

Chimeric antigen receptor (CAR) T‐cell therapy has shown potential in the treatment of PM. This approach involves genetically engineering a patient's T cells to endow them with the capacity to recognize and attack specific tumor antigens. CAR T‐cell therapies for PM have primarily targeted several specific tumor‐associated antigens, including MSLN, placental alkaline phosphatase‐like 2 (ALPPL2), fibroblast activation protein (FAP), and MET. These targets have demonstrated promising efficacy in preclinical animal models [141, 142, 143, 144, 145]. In clinical trials, such as the Phase I trial (NCT02414269), the use of MSLN‐targeted CAR T cells in combination with pembrolizumab achieved an ORR of 63%, with an mOS of 23.9 months and a 1‐year survival rate of 83% in PM patients [146]. A novel multichain DAP (DNAX‐activating protein)‐based CAR T‐cell construct, incorporating a truncated NK cell receptor and DAP12 signaling domain, demonstrated superior cytotoxicity and antitumor efficacy, compared to conventional CAR designs. In a Phase I clinical trial enrolling patients with MSLN‐positive ovarian cancer and mesothelioma, this MSLN‐targeted DAP‐CAR‐T therapy was well tolerated and showed preliminary signs of clinical benefit [147]. Single‐cell transcriptomic profiling revealed transient remodeling of the tumor immune microenvironment and enhanced recruitment of immune cells following CAR T‐cell infusion. These data highlight the potential of CAR T‐cell therapy in treating PM, particularly in improving survival outcomes. Taken together, the primary target for CAR T‐cell therapy in PM remains MSLN, with some progress being made in the exploration of alternative targets such as ALPPL2 and FAP. However, the majority of these targets have yet to advance to clinical stages, highlighting the need for further investigation into novel targets. A recent study has demonstrated that CAR T‐cell therapy targeting sialylated HEG1 protein exhibits promising efficacy and safety in both MM cell lines and xenograft mouse models. These findings underscore the considerable potential of HEG1 as a novel therapeutic target in mesothelioma [148].

While CAR T‐cell therapy has shown initial success in PM, it remains in the early stages of clinical investigation. Potential adverse effects of CAR T‐cell therapy include cytokine release syndrome (CRS) and immune effector cell‐associated neurotoxicity syndrome (iCANS), which are more commonly observed in hematologic malignancies but appear relatively rare and manageable in PM patients [73]. Future research should focus on larger patient cohorts to better understand the interactions between CAR T cells and the TME, as well as explore optimal combination therapy strategies to further enhance efficacy and safety.

### 5.2 Epigenetic Landscapes in PM

Epigenetics plays an increasingly pivotal role in the treatment of PM. Epigenetic mechanisms, including DNA methylation, histone modifications, and non‐coding RNA regulation, have profound effects on the onset and progression of PM.

#### 5.2.1 DNA Methylation

DNA methylation, a key epigenetic regulatory mechanism, has recently emerged as a crucial determinant of the tumor immune phenotype in PM. In a comprehensive study integrating EPIC methylome and transcriptome analyses across 14 PM cell lines of various histological origins, cells were stratified into two methylation‐defined classes—CIMP (hypermethylated) and LOW (hypomethylated)—independent of epithelioid or non‐epithelioid histology [149]. CIMP‐type cells exhibited a transcriptionally immune‐suppressed profile, characterized by extensive silencing of immune‐related genes via hypermethylation. Notably, treatment with the DNA hypomethylating agent guadecitabine reversed these epigenetically silenced immune genes in CIMP cells and further enhanced immune‐favorable signatures in LOW‐type cells. These findings underscore the pivotal role of DNA methylation in shaping the constitutive immune landscape of PM and support the rationale for incorporating epigenetic therapies as a strategy to enhance responsiveness to immune checkpoint blockade.

Several genes have been identified whose methylation status is closely linked to the development of PM. Significant differences in DNA methylation have been observed in the leukocytes of PM patients. For instance, the risk of PM is markedly elevated when low methylation of FKBP5 and high methylation of MLLT1 coincide with substantial asbestos exposure. Such abnormal methylation states may also imply a poorer prognosis, as they are indicative of highly malignant tumor characteristics [150]. Another study, analyzing pre‐diagnostic blood samples from PM patients, identified nine differentially methylated CpGs associated with genes like RAP1A, LHX6, and HOOK2, which are closely linked to early diagnosis and risk assessment for PM [151]. A meta‐analysis has further revealed that the APC gene is significantly hypomethylated in mesothelioma, while CDH1, ESR1, miR‐34b/c, PGR, RARβ, SFRP1, and WIF1 are significantly hypermethylated [152].

KDM4A (lysine‐specific demethylase 4A) is a histone demethylase that primarily targets the methylation marks at lysine 9 (H3K9me3) and lysine 36 (H3K36me3) of histone H3, opening chromatin structures through demethylation, which facilitates gene transcription and plays a crucial role in cancer development [153]. Targeted knockdown and small‐molecule inhibition of KDM4A have been shown to suppress PM growth, and when combined with the BH3 mimetic navitoclax, significantly enhance apoptosis in PM cells [154]. However, research into KDM4A's role in PM remains limited, and the development of clinically applicable targeted drugs requires further investigation.

LSD1 (lysine‐specific demethylase 1) uses FAD as a cofactor to demethylate specific lysine residues on histone H3, particularly H3K4me1/2 and H3K9me1/2 [155]. Inhibition of LSD1 induces a transition of sarcomatoid PM cells to an epithelial phenotype while diminishing their mesenchymal characteristics through activation of the FAK–AKT–GSK3β pathway and the formation of a positive feedback loop between MFGE8 and Snail, thereby significantly increasing their sensitivity to cisplatin‐induced apoptosis [156]. Although research on LSD1 in PM is limited, in other solid tumors, LSD1 inhibition has been found to enhance tumor immunogenicity and T‐cell infiltration in immunogenically “cold” tumors, transforming them into “hot” tumors with active immune responses and significantly improving the efficacy of PD‐1/PD‐L1 ICIs [157]. Furthermore, combining LSD1 inhibition with TGF‐β inhibitors can further enhance CD8+ T‐cell infiltration and release their cytotoxic potential. Thus, a triple therapy comprising LSD1 inhibition, TGF‐β inhibitors, and ICIs may represent a promising strategy, though its efficacy in PM remains to be validated [158].

UHRF1 (Ubiquitin‐Like with PHD and Ring Finger Domains 1) is a multi‐domain protein that recruits DNA methyltransferases to newly synthesized DNA, regulating gene expression by maintaining DNA methylation and modulating chromatin structure [159]. UHRF1 is significantly upregulated in PM and is inversely correlated with patient survival [160]. Knocking down UHRF1 can reverse genome‐wide DNA hypomethylation, inhibiting PM cell proliferation, invasion, and clonogenicity. Similar effects can be achieved using specificity protein 1 (SP1) inhibitors like mithramycin or human double minute 2 (HDM2) inhibitors [161]. These findings suggest that UHRF1 could be a potential therapeutic target, warranting further exploration.

#### 5.2.2 Histone Modifications

EZH2 (Enhancer of Zeste Homolog 2) is the catalytic subunit of the polycomb repressive complex 2 (PRC2), a histone methyltransferase responsible for catalyzing the trimethylation of lysine 27 on histone H3 (H3K27me3). This epigenetic modification leads to gene silencing, particularly in genes involved in developmental regulation. EZH2 plays a critical role in maintaining stem cell self‐renewal and in cancer initiation and progression [162]. Numerous in vitro studies have demonstrated that inhibiting EZH2 can reverse these silencing effects, thereby restoring the activity of tumor suppressor genes and inhibiting tumor growth and progression [163, 164]. Moreover, EZH2 is implicated in regulating immune evasion mechanisms within the TME. Inhibition of EZH2 can upregulate PD‐1 on macrophage surfaces, reducing their cytotoxic effects on PM cell lines, whereas dual inhibition of EZH2 and PD‐1 can restore macrophage immune editing activity [165]. This finding provides a rationale for combining EZH2 inhibitors with ICIs. Clinical trials with the EZH2‐targeted drug tazemetostat are ongoing. An open‐label, single‐arm Phase II study involving 74 patients with relapsed or refractory PM with BAP1 loss showed an ORR of 13%, a PFS of 5.6 months, and an OS of 12.8 months, with no complete responses observed [166]. Although tazemetostat modestly extended survival, the overall efficacy remains limited, warranting further investigation.

HDACs (histone deacetylases) remove acetyl groups from lysine residues on histones, leading to tighter DNA‐histone binding and repression of gene transcription [167]. Panobinostat, an HDAC inhibitor (HDACi), has demonstrated significant antitumor activity in mesothelioma animal models, reducing tumor growth by an average of 62% [168]. Another HDACi, vorinostat (SAHA), enhances the sensitivity of PM cells to cisplatin by downregulating FLIP protein expression [169]. In mesothelioma cell lines with high HDAC expression, such as the Phi and ROB cell lines, SAHA significantly inhibits tumor cell growth, potentially due to mechanisms involving oxidative stress [170]. However, a Phase III clinical study revealed that SAHA monotherapy did not significantly prolong survival, compared to placebo, in patients with advanced or relapsed PM (mOS: 30.7 weeks vs. 27.1 weeks) [171]. Another study found that PM cells with BAP1 loss exhibit downregulation of HDAC2 at the transcriptional level, while HDAC1 is upregulated compensatorily. This imbalance in the ratio of HDAC isoforms renders BAP1‐deficient PM cells more sensitive to HDACi [170]. Additionally, HDACi can modulate the TME in PM. One study reported that another HDACi, valproate (VPA) enhances direct contact between monocytes and tumor cells, promoting tumor cell apoptosis. VPA also induces a phenotypic shift of monocytes from an M2 (immunosuppressive) to a more aggressive M1 phenotype and promotes oxidative stress. The study suggests that combining VPA with ICIs might yield promising therapeutic outcomes, although further research is needed to validate these findings [172].

#### 5.2.3 Non‐Coding RNAs

Both miRNAs and long non‐coding RNAs (lncRNAs) play a significant role in the epigenetic regulation of PM. Aberrant expression of specific miRNAs and lncRNAs is associated with the initiation, progression, and drug sensitivity of PM.

miRNAs are small non‐coding RNAs, approximately 20–24 nucleotides in length, that regulate gene expression by binding to the untranslated regions of target mRNAs, leading to their degradation or translational repression. They play a critical role in the development and progression of various cancers [173].

Several miRNAs have been shown to inhibit PM cell growth and promote apoptosis. miR‐16‐5p, for example, targets and suppresses anti‐apoptotic genes and cell cycle‐related genes, thereby promoting tumor cell apoptosis. In PM cell lines, miR‐16‐5p is significantly downregulated [174]. Additionally, miR‐16‐5p has been associated with chemotherapy resistance and is believed to modulate PD‐L1 expression to some extent [175]. Notably, early clinical studies on miR‐16‐5p have demonstrated good safety, tolerability, and some antitumor activity, although further research is warranted [176]. Other miRNAs with similar functions include miR‐193a‐3p, miR‐137‐3p, miR‐34b/c, and miR‐215‐5p, all of which can inhibit PM tumor cells [174, 177, 178]. Transfecting miR‐34a‐5p into PM cells has been shown to cause cell cycle arrest at the G0/G1 phase, inhibiting proliferation and enhancing the anticancer effects of cisplatin [179]. In another study, researchers delivered miR‐126 to PM cells using exosomes derived from human umbilical vein endothelial cells. They found that miR‐126 inhibited PM cell proliferation and induced apoptosis by modulating angiogenesis‐related genes (such as VEGF and EGFL7) and the IRS1‐mediated signaling pathway [180]. miR‐206 directly targets CDK6 in the RAS signaling pathway, and its introduction into PM cells inhibits tumor growth; this effect is further enhanced when combined with the cyclin‐dependent kinases 4 and 6 (CDK4/6) inhibitor abemaciclib [181]. Recent research has identified miR‐106b as a tumor suppressor by inhibiting cell migration and invasion, potentially mediated by the upregulation of the TCF21 gene [182]. Similarly, miR‐142‐3p downregulates the expression of integrin αV on the cell membrane, affecting the adhesive function of vitronectin in the ECM, thereby inhibiting the invasion and adhesion of PM cells [183].

While most miRNAs are downregulated in PM, some exhibit elevated expression levels and contribute to disease progression. Inhibiting these overexpressed miRNAs using antisense oligonucleotides may offer a promising therapeutic strategy [184]. For example, miR‐182 and miR‐183 promote mesothelioma cell proliferation, invasion, and adhesion via the FOXO1 signaling pathway, and their inhibition significantly impairs these malignant properties [185]. These studies suggest that targeting specific miRNAs may represent a promising therapeutic strategy for PM but further clinical trials are needed to validate this approach.

lncRNAs, a class of non‐coding RNAs longer than 200 nucleotides, can influence gene expression through various mechanisms. It is well established that lncRNAs play critical roles in the initiation and progression of many cancers, including involvement in key processes such as ROS production, genetic alterations (including mutations), epigenetic modifications, and signal transduction pathways [186]. However, research on how lncRNAs affect PM remains relatively limited. Current studies suggest that certain lncRNAs, such as RP1‐86D1.3, GAS5, and MALAT1, may have potential value in diagnosis and prognosis assessment, but these findings require validation with larger datasets [187, 188, 189]. Plasmacytoma variant translocation 1 (PVT1) has been identified as an oncogenic lncRNA implicated in multiple cancers. Recent research indicates that PVT1 promotes PM cell proliferation by enhancing the transcription of Forkhead box M1 (FOXM1) and its downregulation can induce cell cycle arrest at the G2/M phase and inhibit the proliferation and migratory capacity of PM cells [190]. Another lncRNA, LINC00152, is believed to regulate the proliferation and migration of PM cells through interaction with EZH2, providing a theoretical basis for combination therapy [191]. Linc00941 has been shown to bind to the translation initiation factor eIF4G, thereby promoting the selective synthesis of c‐MYC protein and enhancing the expression of key genes involved in translation [192].

### 5.3 Novel Genetic and Molecular Targets in PM Therapy

#### 5.3.1 Genomics Alternations of PM

Current literature identifies several common genetic mutations in PM, including BAP1, NF2, CDKN2A/CDKN2B, and TP53. Unfortunately, most of these mutated genes are tumor suppressors rather than oncogenic drivers, making the development of targeted therapies particularly challenging. As a result, no targeted therapy has yet been approved for PM in clinical practice [193].

BAP1 is a nuclear deubiquitinating enzyme that suppresses tumor growth through its deubiquitination activity. BAP1 is the most frequently mutated gene in PM, with mutation rates ranging from 20% to 60% [194]. The Polycomb Repressive Complex 1 (PRC1) catalyzes the monoubiquitination of lysine 119 on histone H2A (H2AK119ub1), maintaining gene silencing through chromatin modification, which is further reinforced by the PRC2 complex. BAP1 counteracts this by removing the ubiquitin mark, thereby facilitating chromatin opening and gene expression [195]. Given that EZH2 is a key component of PRC2, targeting EZH2 has been proposed as a means to disrupt the cooperative action of PRC1 and PRC2, potentially restoring the tumor‐suppressive function of BAP1 [193]. Furthermore, BAP1 can directly interact with the transcription factor YingYang1 (YY1) to jointly suppress the expression of TNF‐related apoptosis‐inducing ligand (TRAIL) [194]. YY1 is overexpressed in many cancers, and its high expression is associated with poor clinical outcomes and resistance to chemotherapy and immunotherapy [196]. Thus, targeting the BAP1‐YY1 axis may represent a novel therapeutic approach in PM.

**FIGURE 4:**
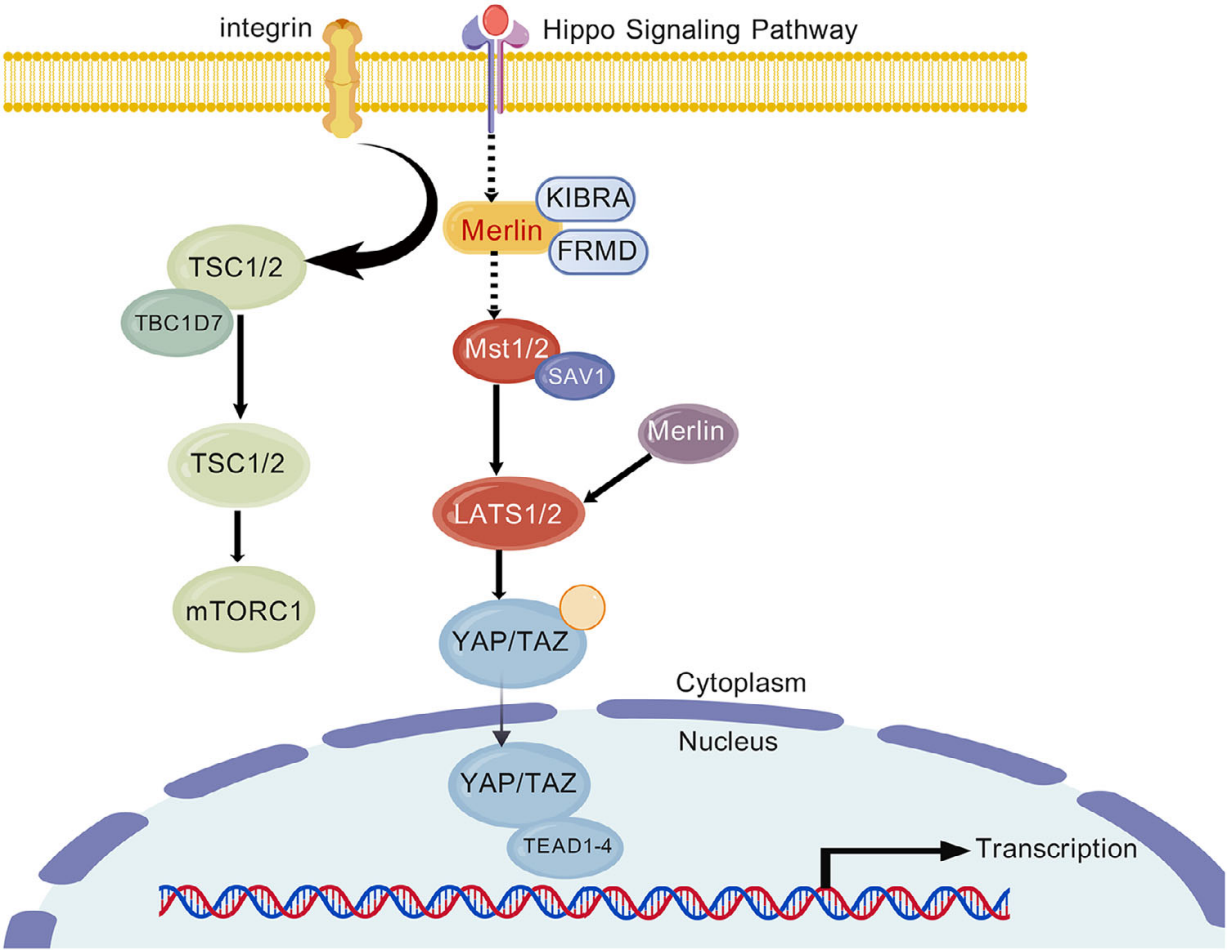
NF2/Merlin regulates the Hippo and mTORC1 signaling pathway in PM, thereby influencing cellular growth and tumor progression.

Mutations in the NF2 gene represent another common genetic alteration in PM, occurring in approximately 20% to 40% of patients [197] (Figure 4). The NF2 gene encodes the protein Merlin (also known as Schwannomin), a key regulator of the Hippo signaling pathway. Loss of NF2 function promotes the nuclear translocation of YAP/TAZ, transcriptional co‐activators that interact with TEA domain family members 1–4 (TEAD1‐4) to form the YAP/TAZ‐TEAD complex, which drives the expression of various oncogenes and supports PM progression [198]. Under normal conditions, the Hippo pathway activates the LATS2 kinase, which phosphorylates and inhibits YAP activity. Another study found that mutations in LATS2 frequently co‐occur with NF2 mutations in PM, resulting in unchecked YAP activity and being commonly associated with poor prognosis [199]. This suggests that targeting the YAP/TAZ‐TEAD complex could be a viable therapeutic strategy for PM. Inhibitors such as SWTX‐143 and IAG933 have demonstrated promising antitumor activity in vitro, supporting their progression to clinical trials [200, 201]. However, most YAP/TAZ‐TEAD inhibitors currently in clinical trials remain in Phase I. While some have shown antitumor efficacy in other solid tumors, their effectiveness in PM has been less encouraging, necessitating further investigation. mTOR (mechanistic target of rapamycin) is another pathway influenced by NF2 mutations. Loss of NF2 function can activate mTORC1 through integrin signaling, promoting tumor cell proliferation and PM cells with NF2 loss exhibit sensitivity to mTOR inhibitors, such as rapamycin [202]. Thus, targeting mTOR may offer another therapeutic avenue for PM patients harboring NF2 mutations. Another research indicates that PM tumors with NF2 loss often display defects in the B‐cell receptor (BCR) signaling pathway and are sensitive to BCR‐ABL/SRC inhibitors [198]. More data are warranted to further confirm its effect.

Mutations in the CDKN2 gene are another common alteration in PM. This gene encodes two tumor suppressor proteins, p16/INK4A and p14/ARF, which are frequently lost in PM tumors. Studies have found that PM patients positive for p14/ARF exhibit higher rates of PD‐L1 expression, suggesting the potential for combined therapeutic approaches in these patients [203]. The p16/INK4A protein prevents cells from progressing from the G1 phase to the S phase by inhibiting the activity of CDK4/6 [204]. Recent in vitro studies have shown that CDK4/6 inhibitors can suppress tumor cell growth by enhancing interferon signaling pathways and the presentation of tumor antigens [205]. Additionally, research evaluating the sensitivity of various PM cell lines to CDK4/6 inhibitors found that 80% of PM samples displayed phosphorylated CDK4 at T172, and these cell lines were sensitive to CDK4/6 inhibitors [206]. Thus, assessing CDK4 phosphorylation status could serve as a predictor of PM sensitivity to CDK4/6 inhibition. These findings support further clinical evaluation of CDK4/6 inhibitors in PM treatment. In a single‐arm, open‐label Phase II MisT2 trial, 26 patients with relapsed, p16/INK4A‐negative PM received 200 mg of abemaciclib, an oral CDK4/6 inhibitor, daily in 21‐day cycles. The DCR at 12 weeks was 54%, and at 24 weeks, it was 23% [207]. Further exploration of novel drugs or combination strategies is needed.

Checkpoint kinase 1 (CHK1) is a key regulator of cell cycle progression, particularly the G2/M checkpoint, and plays an essential role in the DNA damage response by facilitating DNA repair and maintaining genomic integrity [208]. In cancer cells with impaired DNA repair machinery, such as those harboring BAP1 loss or TP53 mutations—common features in PM—CHK1 activity supports survival under genotoxic stress, making it a promising therapeutic target. Recent studies revealed that high CHK1 expression correlates with significantly worse OS in TCGA mesothelioma cohorts [209]. In vitro, CHK1 inhibitors induced dose‐dependent apoptosis, triggered STING pathway activation, and upregulated type I/II interferons, CCL5, and CXCL10, alongside PD‐L1 expression, suggesting an immunostimulatory effect. In vivo, CHK1 inhibition combined with cisplatin markedly suppressed tumor growth, while its combination with anti‐PD‐L1 antibodies extended survival in murine models. These findings indicate that CHK1 targeting not only impairs tumor proliferation but also enhances anti‐tumor immunity, supporting its potential in mesothelioma combination therapy strategies.

While many gene mutations in PM are relatively rare, certain specific mutations exhibit potential clinical significance and therapeutic relevance. For instance, although ALK rearrangements are uncommon in PM, the tyrosine kinase inhibitor crizotinib has shown unexpected efficacy in some patients [210]. Additionally, reports have documented that patients with PM harboring a BRAF V600E mutation experienced significant tumor shrinkage and symptom relief following treatment with vemurafenib [211]. These cases suggest that certain rare genetic mutations in PM, when targeted with corresponding therapies, might yield favorable outcomes, although further clinical data are needed to substantiate these findings.

#### 5.3.2 Signaling Pathway Linked to PM

Upstream and downstream proteins in signaling pathways regulate cellular processes such as growth, division, survival, and death. Aberrant activation of upstream proteins or dysregulated downstream signal transduction can lead to uncontrolled cellular behavior, promoting tumor initiation, progression, and metastasis. Understanding these mechanisms is crucial for the development of targeted cancer therapies.

The PI3K/Akt/mTOR pathway is a key regulator of cell proliferation, survival, and metabolism. In PM, overactivation of the PI3K/Akt/mTOR signaling pathway is considered a major mechanism driving tumor cell proliferation and resistance to apoptosis [212]. In vitro studies have shown that the mTOR inhibitor everolimus, combined with the multi‐target tyrosine kinase inhibitor sorafenib, can significantly inhibit PM growth [213]. However, a Phase II clinical trial evaluating everolimus in relapsed PM patients reported a mPFS of only 2.8 months (95% CI: 1.8–3.4 months), with an overall response rate of just 2% (95% CI: 0%–12%), suggesting that single‐agent everolimus may not be suitable as a standard treatment for advanced PM [214]. Further studies are needed to identify more effective treatment regimens or combination strategies. Another Phase I clinical trial assessed the efficacy and safety of LY3023414, a dual PI3K/mTOR inhibitor, in patients with advanced PM, but only one patient achieved a partial response, and DCR was 43% [215]. This highlights the need for further investigation into its efficacy in a larger patient population and its potential use in combination with other therapies. Fibulin‐3, an ECM protein commonly elevated in the pleural effusions of PM patients, is associated with tumor growth and invasion tissues [216, 217]. Its expression is upregulated in the pleural effusions of PM patients, and it holds potential as a prognostic biomarker for PM. Although Fibulin‐3's diagnostic relevance in PM is lower, compared to mesothelin, its prognostic potential remains substantial [218]. Recent studies have shown that Fibulin‐3 can activate the PI3K/Akt signaling pathway, increasing the expression of genes related to cell adhesion, motility, and invasion. The combined use of a PI3K inhibitor and anti‐Fibulin‐3 antibodies significantly inhibited tumor cell proliferation and growth, suggesting that future studies targeting the PI3K/Akt/mTOR pathway should consider combination therapies [219].

The Hedgehog signaling pathway (HP) plays a pivotal role in embryonic mesothelial development; however, it typically remains inactive in most adult tissues, including the mesothelium, and is only reactivated in specific progenitor cells [220]. In PM, the HP is aberrantly reactivated in some patients, and its inhibition has been shown to reduce the growth of PM cells [221]. The activation of the HP relies on the binding of Hedgehog ligands to the PTCH1 receptor, which relieves the inhibition on SMO, ultimately leading to the nuclear translocation of GLI family proteins and the induction of target gene expression [222]. In PM, the dysregulated activation of the HP is associated with the tissue repair process following damage, and the elevated expression of SMO, Shh, and GLI1 correlates significantly with poor patient prognosis [221, 223, 224, 225]. Genomic analysis of tumor samples from 1113 PM patients revealed mutations in HP genes (such as PTCH1 and SUFU), though these mutations were rare (PTCH1 at 1.2%, SUFU at 0.8%) [226]. Notably, research targeting the HP signaling pathway has largely not progressed to clinical trials. Although in a rat mesothelioma model, the SMO inhibitor vismodegib significantly reduced tumor volume and growth, and lowered the expression of HP target genes such as GLI1, HHIP, and PTCH1, the two available Phase I clinical trials of vismodegib and sonidegib failed to demonstrate clinical benefit, potentially due to the lack of genotype‐based patient selection [227, 228, 229, 230]. In summary, research on the HP signaling pathway in PM remains sparse, and further exploration of the key molecular regulators of this pathway, such as SMO and GLI, is warranted.

The ECM plays a pivotal role in cellular expansion and maturation, with heparan sulfate proteoglycans playing a significant role in the regulation of intercellular adhesion and signal transduction [231]. Heparanase, the primary enzyme responsible for the degradation of intercellular heparan sulfate, is markedly upregulated in malignant tumors [232]. Individuals with elevated levels of heparanase immunoreactivity exhibit shorter survival times, whereas patients with lower heparanase levels tend to have prolonged survival [233]. Heparanase inhibitors, such as defibrotide and pixatimod (PG545), have shown superior efficacy in inhibiting tumor progression, compared to cisplatin [233]. These inhibitors significantly extended survival in preclinical murine models of mesothelioma. A novel dicarboxylated oxy‐heparin derivative XII is a heparinase‐targeting agent, demonstrating multifaceted antitumor efficacy and improved survival outcomes across preclinical models of pancreatic cancer, breast cancer, mesothelioma, and myeloma, mediated via concerted inhibition of enzymatic activity, expression, and cellular trafficking [234]. A recent study highlighted that a three‐dimensional culture environment reshapes the transcriptome of PM cells, particularly upregulating genes associated with ECM assembly, such as COL1A1 and COL5A1, suggesting that the three‐dimensional growth environment may influence tumor cell tensile structure by altering ECM stiffness [235]. The formation of PM tumors is driven by a chronic inflammatory response induced by the initially produced pro‐inflammatory asbestos fibers and is accompanied by extensive fibrosis, a primary driver of pain and respiratory dysfunction. In mesothelioma, numerous cell types participate in ECM remodeling and fibrosis, with CAFs playing a central role, being closely linked to tumor progression and prognosis [11]. PM cells can activate CAFs by secreting cytokines and growth factors, promoting a pro‐fibrotic, pro‐inflammatory, and chemotactic phenotype that accelerates PM cell growth, resistance, and survival [236]. Targeting the signaling interactions between PM cells and CAFs, such as using the SRC family kinase inhibitor saracatinib, effectively prolonged survival in mouse models and demonstrated superior efficacy, compared to conventional standard treatments, such as cisplatin/pemetrexed combination therapy [237].

#### 5.3.3 Mesothelin (MSLN)

Mesothelin (MSLN) is a membrane‐bound extracellular protein and a cancer‐associated antigen that binds to mucin 16. It is expressed at low levels in normal tissues but at high levels in PM, where it is correlated with poor survival [238]. MSLN also modulates the immune microenvironment of PM. Studies have found that high MSLN expression is associated with the epithelioid PM subtype and a high density of CD8+ T cells, CD68+ macrophages, and type I collagen fibers, whereas low MSLN expression is linked to tumor necrosis and nuclear Grade 1 (low malignancy) [239]. Current MSLN‐targeted therapeutic strategies encompass a range of approaches, including chimeric monoclonal antibodies (such as amatuximab), antibody‐drug conjugates (ADCs; such as anetumab ravtansine [AR], BMS‐986148, and BAY2287411), immunotoxins (such as SS1P and LMB‐100), cancer vaccines (such as the Listeria‐based vaccine expressing MSLN), and CAR T‐cell immunotherapy [238].

Amatuximab (MORab‐009) is a chimeric IgG1κ monoclonal antibody that targets the extracellular domain of MSLN. Its antitumor mechanism exhibits dual characteristics: On the one hand, it mediates antibody‐dependent cellular cytotoxicity (ADCC) by binding to MSLN, thereby activating immune effector cells such as NK cells to kill tumor cells; on the other hand, it competitively blocks the ligand‐receptor interaction between MSLN and MUC16 (CA125), thereby inhibiting tumor cell adhesion and metastatic behaviors [240]. In a preliminary Phase II single‐arm trial (NCT00738582), the combination of amatuximab with pemetrexed/cisplatin demonstrated initial clinical activity in patients with unresectable PM, achieving an mOS of 14.8 months [241]. However, subsequent randomzed, double‐blind, placebo‐controlled Phase II trials (ARTEMIS, NCT02357147) were prematurely terminated due to failure to meet predefined efficacy endpoints. Notably, mechanistic studies revealed that the antibody may form immune complexes by binding to free MUC16 in patient serum, which significantly diminishes Fcγ receptor‐mediated ADCC, thereby affecting its therapeutic efficacy [242]. Given these pharmacodynamic limitations and the potential for off‐target effects, the clinical development of this agent is largely at a standstill. Nevertheless, recent research suggests that engineering the Fc domain or developing bispecific antibodies may offer promising strategies to optimize the therapeutic window for MSLN‐targeted treatments [243].

ADCs achieve tumor‐specific cytotoxicity by linking antibodies targeting MSLN with cytotoxic agents, combining targeting precision with potent cellular toxicity. AR, a representative ADC, is composed of a humanized MF‐T antibody conjugated to the microtubule inhibitor DM4 (ravtansine). It exerts its cytotoxic effect by disrupting microtubule function and demonstrates bystander effects on adjacent MSLN‐negative cells [244]. Phase I/II trials (NCT01439152) revealed a DCR of 75% in patients with PM, but subsequent randomized Phase II trials indicated that it failed to significantly improve PFS as a second‐line treatment [245]. Current research is now focused on exploring combination therapies with pemetrexed/cisplatin chemotherapy or pembrolizumab (anti‐PD‐1). A recent study suggests that soluble MSLN (sMSLN) in plasma may bind to anetumab, impairing its delivery to tumor tissues and thus diminishing its efficacy. Therapeutic plasma exchange (TPE) effectively clears sMSLN, potentially restoring the activity of MSLN‐targeted therapies [246]. Another ADC, BMS‐986148, employs tubulysin as its payload. Its Phase I/II trial (NCT02341625) demonstrated that combination with nivolumab resulted in an ORR of 31%, significantly higher than the 4% achieved with monotherapy, with some patients maintaining a durable response for up to 9 months [247]. Current evidence suggests that while MSLN‐targeted ADCs (such as AR and BMS‐986148) show limited monotherapy efficacy, combination strategies with chemotherapy or ICIs significantly enhance clinical outcomes. Additionally, innovative delivery systems based on physical targeting (APMS) and biological carriers (EDVs) offer promising strategies to overcome the limitations of traditional treatments [248, 249]. Recent research employing high‐throughput screening platforms to optimize anti‐MSLN monoclonal antibody variants—comprising over 300 single‐chain variable fragments and more than 50 IgG complementarity‐determining regions (CDRs)—has identified key CDR residues crucial for binding. Furthermore, hydrophilic optimization has substantially improved antibody solubility and ADC efficacy in murine gastric and pancreatic cancer models [250]. Moving forward, future research should focus on optimizing payload release kinetics, addressing tumor heterogeneity, and utilizing biomarkers for precise patient stratification.

Compared to other MSLN‐targeted therapies, CAR T‐cell therapy offers several potential advantages, including long‐term immune surveillance and a reduced likelihood of tumor recurrence [251, 252]. Unlike other therapies, anti‐MSLN CAR T cells do not react with SMRP (mesothelin‐related protein) and specifically induce cytotoxic effects only on membrane‐bound MSLN, thereby enhancing both the specificity and safety of the treatment [253]. Early clinical trial results have shown that CAR T cells exhibit limited persistence within the patient's body, as well as insufficient tumor infiltration, which may account for their limited efficacy [254, 255]. Researchers have made efforts to enhance the therapeutic outcomes by optimizing the delivery methods and functional properties of CAR T cells. Traditional intravenous (IV) administration is hindered by physiological barriers, leading to poor accumulation of CAR T cells at the tumor site. In contrast, local delivery methods, such as intrapleural injection, significantly increase the concentration of CAR T cells within the TME, thereby enhancing their antitumor efficacy [141, 146]. Furthermore, optimizing CAR T‐cell functionality through genetic engineering represents a critical strategy for improving treatment outcomes. For instance, engineering anti‐MSLN CAR T cells to express the chemokine receptor CCR2 enhances their migratory capacity toward the tumor site. Additionally, the use of CXCR4 antagonists further facilitates their accumulation within the tumor region [256, 257]. CAR T cells targeting MSLN combined with secretion of PD‐1 nanobodies have shown preliminary efficacy in clinical trials, with some patients achieving prolonged survival [258]. Combining MSLN‐targeted CAR T‐cell therapy with ICIs may also represent a viable therapeutic strategy [146]. Currently, studies involving MSLN‐targeted CAR‐T therapy and PD‐L1 combination therapies are in Phase I clinical trials, and further data are needed to support these approaches.

## 6 Conclusion and Prospects

The current therapeutic strategies for PM integrate chemotherapy, anti‐angiogenic agents, and immunotherapy, yet these combined approaches yield only modest survival benefits due to persistent challenges such as therapeutic resistance and tumor heterogeneity. Despite promising preclinical discoveries, the translation of molecular targets into clinically validated therapies remains uncertain, reflecting the complex pathobiology of PM that demands systematic investigation. Recent advances have illuminated potential mechanistic dimensions, such as dynamic crosstalk between stromal and immune cells within the TME, subtype‐specific metabolic reprogramming, and epigenetic dysregulation. Collectively, these insights delineate a pressing translational challenge—harnessing multidimensional mechanistic understanding to develop clinically impactful interventions.

Emerging technologies, including single‐cell spatial omics and AI‐driven predictive modeling, are redefining target discovery by enabling the identification of key molecular drivers within intricate biological networks. Concurrently, advanced preclinical models that recapitulate disease evolution are enhancing the predictive validity of therapeutic screening.

Future breakthroughs in PM management will hinge on the convergence of multidisciplinary expertise. By elucidating core oncogenic mechanisms, leveraging innovative platforms for therapeutic optimization, and establishing rapid translational pipelines, the field may evolve from incremental survival gains toward achieving durable disease control. This integrative framework, bridging fundamental discovery with clinical validation, promises to redefine therapeutic goals and outcomes for patients with this recalcitrant malignancy.

## Author Contributions

L.Z. drafted the manuscript. M.H. designed and revised the manuscript. All authors have reviewed and endorsed the final manuscript.

## Ethics Statement

The authors have nothing to report.

## Conflicts of Interest

The authors declare no conflicts of interest.

## Data Availability

The authors have nothing to report.
